# Chronic cannabis use and sleep architecture: a cross-sectional analysis of polysomnography outcomes in a sleep-clinic cohort

**DOI:** 10.1093/sleep/zsaf396

**Published:** 2025-12-18

**Authors:** Rob Velzeboer, Sabrina Wei, Wayne W K Lai

**Affiliations:** Clinical Research Department, Tranq Sleep Care, Kelowna, BC, Canada; Department of Interdisciplinary Studies, Faculty of Arts, University of British Columbia, Vancouver, BC, Canada; Clinical Research Department, Tranq Sleep Care, Kelowna, BC, Canada; Department of Psychology, Faculty of Arts, University of British Columbia, Vancouver, BC, Canada; Clinical Research Department, Tranq Sleep Care, Kelowna, BC, Canada; Department of Medicine—Neurology, Faculty of Medicine, University of British Columbia, Vancouver, BC, Canada

**Keywords:** chronic, cannabis, THC, polysomnography, sleep architecture, sleep fragmentation, sleep staging

## Abstract

**Study Objectives:**

Cannabis is widely self-administered as a sleep aid, yet objective evidence from large polysomnography cohorts remains scarce. We assessed whether long-term daily cannabis use is associated with alterations in overnight sleep architecture at a Canadian sleep clinic.

**Methods:**

We retrospectively analyzed overnight polysomnography studies from 1449 adult sleep clinic patients. Exposure was chronic cannabis use, defined as ≥daily consumption for ≥1 year (*n* = 151). Never-users (*n* = 1298) served as the reference group. Nine polysomnography outcomes—total sleep time, sleep onset latency, wake after sleep onset, sleep efficiency, rapid eye movement (REM) latency, and N1, N2, N3, and REM sleep (presence and duration)—were modeled with outcome-appropriate regressions adjusted for 28 demographic, lifestyle, comorbidity, medication, and sleep-related covariates.

**Results:**

Chronic cannabis use was associated with higher wake after sleep onset (β = 21%; 95% CI 6.7% to 37.2%), lower sleep efficiency (β = −3.8%; 95% CI −6.6% to 1.0%), and elevated N1 (β = 2.8 percentage points [pp]; 95% CI 0.3 to 5.6 pp). Nominally, total sleeping time was lower among cannabis users (β = −3.3%; 95% CI −6.3% to 0.3%). Effect directions and magnitudes persisted across sensitivity analyses.

**Conclusions:**

Among sleep-clinic patients, long-term daily cannabis use was associated with greater nocturnal wakefulness. Given that most participants had moderate-to-severe sleep apnea, findings should be interpreted with caution. Studies detailing dose, timing, and cannabinoid composition are needed to clarify causality and clinical relevance.

**Statement of Significance**

Cannabis is frequently used to manage sleep problems, yet its long-term effects on sleep architecture remain uncertain. This study provides the largest clinic-based assessment to date, linking chronic daily use to objectively measured increases in nocturnal wakefulness among sleep clinic patients primarily referred for sleep apnea, suggesting that habitual cannabis use may fragment sleep. These findings raise important questions for clinicians and researchers, given the widespread use of cannabis as a sleep aid. Longitudinal and experimental studies are needed to clarify how dose, timing, and cannabinoid profile influence sleep and to explore downstream consequences for cognition, mood, and long-term health. Clarifying these pathways will guide patient counseling, therapeutic decisions, and public policy as legalization and social acceptance continue to expand.

## Graphical Abstract

**Graphical Abstract:**
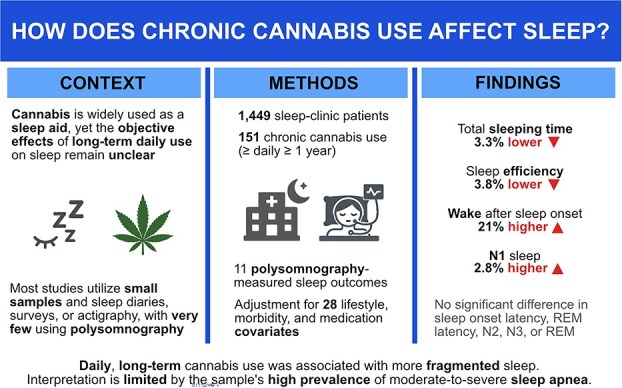

## Introduction

A growing number of North Americans use cannabis to manage poor sleep [1], as the substance has become increasingly available by legal means across Canada and in most jurisdictions in the United States [2]. In fact, some surveys show that sleep is the most commonly reported symptom that cannabis users target, with as many as 85 percent of medical cannabis users, as well as a substantial proportion of recreational users, reporting improvements [3, 4]. Although commonly perceived as a benign or beneficial drug due to its effects on pain and certain neurological disorders [5], cannabis use has been associated with poor mental health [6] and a range of potentially negative effects on cognition and brain function [7].

Sleep plays a crucial role in these functions [8], yet the precise effects of cannabis on sleep remain unclear. Studies utilizing subjective measures such as sleep satisfaction scales frequently show self-reported sleep improvements, particularly among patients with pain symptoms [9, 10]. Those that collect data through objective polysomnography (PSG)—the gold standard for sleep studies [11]—on the other hand, have shown largely inconsistent or null outcomes on key sleep parameters [9, 12, 13].

Cannabis is generally believed to help users fall asleep faster [14], which is thought to be due to the sedative properties of tetrahydrocannabinol (THC), where cannabidiol (CBD) may modulate these effects [15]. Its use has also been consistently associated with a reduction in rapid eye movement (REM) sleep [12, 16, 17], which is important for brain function [18, 19], though this association has not been consistently replicated in modern low-dose studies [13, 20–23]. The existing evidence from clinical trials is marred by small sample sizes and a confluence of confounding factors, such as poor prior use reporting, different medical conditions, subjective metrics, and variation in cannabinoid composition and dosing, that complicate interpretation [9, 13].

A key challenge lies in the diversity of cannabis use patterns. The cannabis delivery system—often smoked, vaporized, and orally ingested, but also consumed through oromucosal spray and topical-transdermal application [24]—may differentially affect sleep outcomes due to differences in bio-availability and metabolite formation as well as altered onset, peak, and duration of cannabinoid exposure [25, 26]. Although nearly all of the clinical literature has focused on oral intake [9, 10], the vast majority of cannabis users report smoking or vaporization as their preferred method [27].

Further, the cannabis plant itself contains over 60 cannabinoids [28], the relative ratios of which are likely to play an important role. The aforementioned THC has been most consistently associated with improvements in sleep across review articles [9, 29–31] and early studies suggest cannabinol (CBN) may be beneficial as well [32, 33], whereas evidence for CBD remains poor and largely unsupported [29, 31]. It has also been suggested that improvements in sleep may be partly or even fully mediated by cannabis’ effects on pain or other medical symptoms [14].

Critically, cannabis’ effects on sleep appear to differ significantly depending on prior use. Frequent users may develop tolerance to some of cannabis’ effects and experience withdrawal symptoms when they stop using due to dependence [34]. Indeed, frequent users often require cannabis in order to have a good night’s sleep [35]. Accordingly, the effects of acute administration likely differ from those of chronic use. How frequent cannabis use affects sleep in the long term remains poorly understood, with observational studies conducted with frequent long-term users generally showing no significant differences in terms of total sleeping times (TSTs), sleep onset, or sleep staging, including REM sleep [36–38].

However, these analyses have excluded the types of clinical or comorbid users most likely to experience altered sleep physiology: subjects include healthy young men volunteering to smoke self-supplied cannabis in laboratory settings [36], long-term “functional” users screened to exclude medical or psychiatric conditions [37], and a convenience sample of healthy university students without sleep disorders [39]. Further, small samples and the lack of control for important confounders such as morbidities and medication use limit the generalizability and validity of these findings. A Canadian sleep clinic study did control for many such confounders, but based its findings on just seven cannabis-using patients within a subgroup of 613, or about 1 percent of its sample [40], far below the estimated prevalence of use in Canada of ~11 percent [41], thereby limiting the generalizability of its findings.

Because self-medication for sleep tends to progress toward higher frequency and dose [42, 43], elevating problem risk and altering sleep outcomes [44], this study focuses on chronic daily users to examine the long-term physiological impact of sustained cannabis exposure on sleep. It leverages a large polysomnographic dataset from a Canadian sleep clinic, using standardized cannabis use reporting and comprehensive adjustment for relevant confounders, including substance use, medication use, and relevant health conditions, to provide a clearer understanding of the long-term physiological effects of sustained cannabis exposure on objectively measured sleep architecture.

## Materials and Methods

### Study design and participants

We conducted a cross-sectional study at an accredited Canadian sleep clinic to compare objective sleep architecture outcomes between patients who reported regular cannabis use and those who did not. These patients were referred for sleep disturbances by their general practitioner to consult with a sleep specialist. The analytic sample was derived from patients who underwent overnight PSG and completed an intake questionnaire detailing substance use, comorbidities, and lifestyle behaviors. Further, a list of active prescriptions was included for each patient. PSG results were scored by registered polysomnographic technologists following *American Academy of Sleep Medicine* standards and were interpreted by a licensed sleep physician.

We manually collected the data of patients who presented to the clinic between November 2022 and January 2024. This data was extracted from PSG reports, active prescription and morbidities lists, and medical office administrator patient “lifestyle” notes, which include self-reported substance use patterns for all patients. Data of titration studies, patient studies with less than 240 minutes of TST [45, 46], and from patients who used cannabis occasionally (<1 year and/or <once daily) was not collected. Occasional users were excluded because infrequent exposure is unlikely to produce physiological tolerance or neuroadaptation relating to sleep [47, 48], and their polysomnographic outcomes would therefore be expected to resemble those of non-users, obscuring the effects characteristic of the chronic use patterns under study.

A total of 1449 patient records were included, 151 of whom met the cannabis use criterion. No instructions were given to patients to abstain from cannabis use prior to their PSG, but patients were asked to let their sleep technologist know that they had used it. The registered polysomnographic technologists who scored the sleep studies were blinded to participants’ cannabis use status.

Ethics approval for the study was granted by the University of British Columbia’s Clinical Research Ethics Board in February of 2024 (H23-01461). The data that support the findings of this study are not publicly available due to privacy and ethical restrictions involving identifiable patient health information (e.g. precise age, morbidity, and medication information). Analytic code used in the primary and sensitivity analyses is provided in the supplementary materials. Additional summary data or code may be made available from the corresponding author upon reasonable request.

### Variable specification

The primary exposure was chronic cannabis use. This is usually defined in the literature as daily or near daily cannabis use continued over the course of several years [34], while cannabis naïve subjects are usually characterized by no prior use of cannabis at all [49, 50], or no use in the past 3 years or more [51]. We thus characterized our exposed group, patients who chronically use cannabis, as those who used at least once daily for at least 1 year (exposure = 1), while our control group was comprised of patients who reported never having used it (exposure = 0).

Cannabis use frequency, duration, and type were collected. However, because documentation varied in phrasing and precision (“smokes, vapes and take edibles 2 to 3 times daily, for 4 years,” “smokes a combination of THC:CBD once a day, since 1986”, “daily, long-term use”), only entries that included a specific number of grams or joints smoked per day (*n* = 69) and/or specific use durations (*n* = 120) were included for analysis. Although cannabis and joint potency are highly variable [52, 53], following empirical cannabis dose conversion approaches [54], we assumed a gram of cannabis contains 200 mg of THC, and a joint 100 mg of THC, based on recent Canadian and British Columbia-specific cannabis use analyses [55, 56]. These were converted to Standard THC Units (STUs), as per STU use recommendations, for descriptive statistics [57, 58]. Use durations were charted as both total years of use and calculated as a percentage of adult life (use from 18 years old onwards).

Outcomes included 11 standard variables collected through PSG: macrostructural sleep measures (TST, sleep efficiency [SE], sleep onset latency [SOL], wake after sleep onset [WASO], REM latency), and stage-specific presence (N3 and REM), and durations (N1%, N2%, N3%, REM%).

Covariates were selected based on clinic data availability and theoretical relevance. For demographic variables these included sex [59], age [60], and body mass index [61, 62]. For lifestyle factors, engagement in night shift work [63], daily alcohol use [17, 64], and nicotine use [17] were coded and included. For comorbid medical and psychiatric conditions, cardiovascular [65], endocrine [66], metabolic [67], gastrological [68], neurological [69], pain-related [70, 71], psychiatric [72, 73], and respiratory disorders were coded. For concurrent use of pharmacologic agents affecting sleep, stimulants/wake-promoting agents [74], sedatives/hypnotics [75], antidepressants [76], antipsychotics [77], beta-blockers [78], corticosteroids [79], diuretics [80], antihistamines [81], dopaminergic agents [82], opioids [83], and non-opioid pain medications were categorized and included [76]. Last, for sleep-related factors, apnea-hypopnea index (AHI) [84], presence of parasomnias [85], and periodic limb movements during sleep (PLMs) were recorded [86]. Given the retrospective and observational nature of the dataset, PLMs, AHI, and parasomnias were treated as control variables rather than outcomes, as these conditions represented pre-existing sleep disturbances that contributed to referral and diagnosis.

### Statistical analysis

All analyses were conducted in R (version 4.5.1) [87]. Baseline characteristics were summarized as mean ± *SD*. Between-group imbalances were quantified using absolute standardized mean differences (SMD) [88], where an SMD ≥ 0.20 was considered indicative of a meaningful difference. SMDs were calculated both before and after adjustment for demographics.

To estimate the association between chronic cannabis use (defined as ≥daily use ≥1 for year vs. never) and each polysomnographic outcome, we fitted covariate-adjusted regression models tailored to the distribution of each outcome. Model type and transformation strategy were selected based on diagnostic evaluations of skew, boundedness, zero inflation, and heteroskedasticity. Right-skewed time-based macro-architectural metrics (TST, SOL, WASO, REM latency, and sleep efficiency) were analysed on a log scale using ordinary least-squares models. Proportion-based outcomes (N1 and N2) were modeled using quasi-binomial generalized linear models with a logit link. Because N3 and REM durations were absent in a subset of individuals, we modeled their presence using logistic regression, and among those with observed values, stage durations were logit-transformed and analyzed using ordinary least squares (OLS). Dirichlet regression was also explored to model the joint distribution of N1%, N2%, N3%, and REM%, but demonstrated poor overall fit. For the full regression equation see Supplementary Appendix 1.

All models adjusted for the same set of demographic, lifestyle, morbidity, medication, and sleep-related variables, with heteroskedasticity-consistent robust standard errors applied to account for heteroskedasticity and the false discovery rate controlled through the Benjamini–Hochberg (BH) procedure. Outcome coefficients were back-transformed to yield marginal effects: percent changes for log-transformed outcomes and absolute percentage-point differences for proportion-based models. Missing values were not observed in PSG studies, as only studies containing valid recordings of all 11 outcomes were collected. A total of 14 out of 1449 patients (1 percent) reported no morbidities, and all patients listed at least one medication.

Exploratory analyses included stratification by sex (male vs female), AHI (none <5, mild 5−<15, moderate 15−<30, severe ≥30), and age (18–34, 35–49, 50–64, 65+), and the testing of interaction terms for these variables. In these models, BH was applied per family: 22 tests for sex, 44 for age, and 44 for AHI. Further, dose–response analyses were performed with cases where reliable data was available, and, given limited data, were treated as exploratory as well.

### Model diagnostics and interpretation

Model diagnostics were performed for each fitted model. For log-transformed OLS models, normality and heteroskedasticity were assessed via residual Q–Q plots, half-normal envelope plots, and the studentized Breusch–Pagan test. For fractional-logit models, model fit was assessed using deviance residual plots and dispersion (φ) estimates. Multicollinearity was monitored via variance inflation factors (VIF), while influence diagnostics included studentized residuals (Bonferroni-adjusted), Cook’s distances (threshold >4/*n*), and hat values (>2 k/*n*). The Ramsey regression equation specification error test (RESET) test (type = “regressor”) was applied across all models to assess global model misspecification. For stage presence models for N3 and REM, model calibration was assessed with Hosmer–Lemeshow (HL) tests and discriminative ability by calculating the area under the received operating characteristic curve (ROC-AUC).

Robustness was evaluated through several sensitivity analyses: (1) re-estimation after winsorising and trimming outliers beyond ±3 *SD*; (2) parsimonious adjustment using minimal confounder sets (3 and 7 variables) based on correlation matrices (see Supplementary Table S1); (3) block exclusion of medication and morbidity variables, separately and jointly; and (4) propensity-score matching (PSM) with 1:1 nearest-neighbor matching (caliper = 0.1 *SD* of the logit) on all baseline covariates, followed by re-estimation of primary models. To assess robustness for sleep staging models, models were re-run while holding TST, SE, and WASO constant.

## Results

### Sample characteristics

Sample characteristics are presented in Table 1. Compared to cannabis-naïve patients, individuals using cannabis were more likely to be male (SMD = 0.251), younger (SMD = −0.595), and showed higher rates of nicotine (SMD = 0.866), sedative (SMD = 0.244), antidepressant (SMD = 0.219), antipsychotic (SMD = 0.355), and opioid use (SMD = 0.207), as well as a higher rate of psychiatric disorders (SMD = 0.418). Cardiovascular disease (SMD = −0.386) and the use of betablockers (SMD = −0.205) and diuretics (SMD = −0.289) were less common among individuals using cannabis. Individuals using cannabis showed higher N1 stage sleep (SMD = 0.262).

**Table 1:** Sample characteristics and SMDs

		Cannabis		Non-cannabis			
		*n* = 151		*n* = 1298			
		**Mean**	***SD***	**Mean**	***SD***	**SMD**	(Adjusted^*^)
Demographic/lifestyle							
	Female	39.7%		52.2%		−0.251	NA
	Age	45.8	±14.5	55.0	±15.6	−0.595	NA
	Body mass index	32.1	±7.8	31.6	±7.3	0.070	(0.005)
	Nightshift worker	3.7%		5.3%		−0.086	(−0.117)
	Daily drinking	10.3%		9.0%		0.045	(0.068)
	Nicotine use	0.57	±0.42	0.26	±0.35	0.866	(0.902)
Medications							
	Stimulants	6.3%		3.7%		0.140	(0.013)
	Sedatives	43.4%		30.7%		0.244	(0.301)
	Antidepressants	46.4%		35.7%		0.219	(0.176)
	Antipsychotics	12.6%		4.1%		0.355	(0.404)
	Betablockers	4.4%		9.6%		−0.205	(−0.102)
	Corticosteroids	1.2%		2.1%		−0.099	(−0.050)
	Antihistamines	3.2%		2.2%		0.081	(0.072)
	Dopaminergic agents	4.8%		7.5%		−0.123	(−0.104)
	Opioids	14.7%		8.2%		0.207	(0.286)
	Diuretics	7.3%		16.6%		−0.289	(−0.139)
	Non-opioid pain meds	25.2%		21.9%		0.078	(0.169)
Comorbidities							
	Cardiovascular	39.1%		58.0%		−0.386	(−0.216)
	Endocrine	21.9%		27.4%		−0.130	(0.015)
	Metabolic	8.0%		10.3%		−0.080	(0.041)
	Gastrological	21.2%		26.7%		−0.130	(−0.017)
	Neurological	13.9%		17.3%		−0.092	(−0.051)
	Pain-related	21.9%		17.3%		0.116	(0.215)
	Psychiatric	59.6%		39.1%		0.418	(0.381)
	Respiratory	9.3%		13.3%		−0.128	(−0.041)
Sleep-related							
	Apnea Hypopnea Index	34.3	±31.8	33.4	±27.9	0.032	(0.088)
	Periodic limb movements	11.7	±18.5	14.5	±23.6	−0.123	(−0.026)
	Parasomnias	2.4%		1.3%		0.117	(0.118)
Sleep duration							
	TST	354.7	±56.1	355.2	±58.8	−0.009	(−0.182)
	Sleep efficiency	77.9	±10.2	77.4	±11.4	0.046	(−0.167)
	Sleep onset latency	26.1	±27.7	25.9	±26.1	0.005	(0.080)
	Wake after sleep onset	75.6	±46.0	79.5	±50.9	−0.076	(0.111)
Sleep staging							
	REM latency	171.6	±105.6	166.0	±97.3	0.057	(0.108)
	N1%	15.7	±14.2	12.8	±10.5	0.262	(0.308)
	N2%	58.7	±14.9	60.9	±13.1	−0.165	(−0.084)
	N3%	11.3	±10.8	11.7	±10.2	−0.035	(−0.127)
	REM%	14.3	±6.9	14.6	±7.7	−0.042	(−0.123)

^*^Adjusted for age and sex; SDM = Standardised mean difference.

After adjusting for age (SMD = −0.595) and sex (SMD = 0.251), differences in nicotine use (SMD = 0.902), antipsychotic use (SMD = 0.404), psychiatric conditions (SMD = 0.381), N1% (SMD = 0.308), sedative use (SMD = 0.301), opioid use (SMD = 0.286), and cardiovascular conditions (SMD = −0.216) remained significant, while differences in diuretic (SMD = −0.139), betablocker (SMD = −0.102), and antidepressant use (SMD = 0.176) fell below the 0.2 threshold. After adjustment, pain conditions (SMD = 0.215) showed to be more common among cannabis users.

### Primary analysis

Table 2 presents the fully adjusted regression model controlling for all 28 covariates. After BH correction, cannabis use was associated with significantly higher WASO (21%, ~16.6 minutes), significantly lower sleep efficiency (~3.8%), and elevated N1% (2.8 percentage points). Nominally, cannabis use was associated with lower TST (−3.3%, ~−11.8 minutes). No significant differences were observed for SOL, REM latency, N2%, N3%, N3 presence, REM%, or REM presence. For the full table including back-transformed outcome coefficients see Supplementary Table S2.

**Table 2:** Primary model outcomes

		Model	β (95% CI)	*P* (q)	%/pp
Sleep quantity and efficiency					
	TST	OLS (log-transformed)	−0.034 (−0.063 to −0.005)	.023 (0.062)	−3.3%
	Sleep efficiency	Quasibinomial GLM (logit)	−0.160 (−0.270 to −0.048)	.005 (0.018)	−3.8%
Transitions and fragmentation					
	Sleep onset latency	OLS (log-transformed)	0.012 (−0.165 to 0.189)	.894 (0.894)	+1.2%
	Wake after sleep onset	OLS (log-transformed)	0.191 (0.070 to 0.311)	.002 (0.012)	+21.0%
	REM latency	OLS (log-transformed)	0.028 (−0.081 to 0.136)	.619 (0.783)	+2.8%
Sleep staging					
	N1%	Quasibinomial GLM (logit)	0.223 (0.080 to 0.364)	.002 (0.012)	+2.8 pp
	N2%	Quasibinomial GLM (logit)	−0.038 (−0.138 to 0.062)	.458 (0.783)	−0.9 pp
	N3%	OLS (logit-transformed)	−0.149 (−0.378 to 0.080)	.202 (0.445)	−1.6 pp
	N3—presence	Logistic regression	−0.116 (−0.635 to 0.429)	.668 (0.783)	NA
	REM%	OLS (logit-transformed)	−0.025 (−0.159 to 0.109)	.712 (0.783)	−0.3 pp
	REM—presence	Logistic regression	−0.237 (−1.147 to 0.821)	.632 (0.783)	NA

q, Benjamini–Hochberg adjusted; OLS = Ordinary Least Squares; GLM = generalized linear model; pp = percentage points.

**Figure 1:**
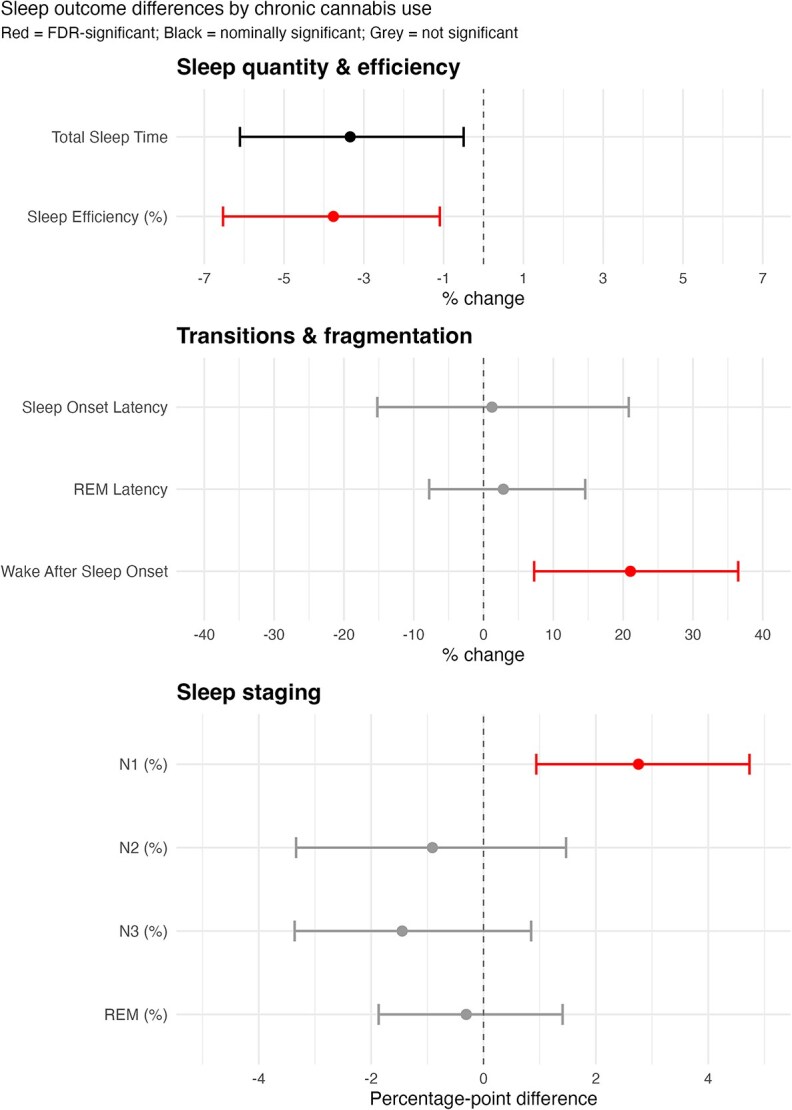
Adjusted differences in sleep architecture between cannabis users and non-users.

Figure 1 depicts covariate adjusted estimates and 95% CIs for duration-based outcomes. Significant false discovery rate (FDR)-corrected differences (WASO, sleep efficiency, N1%) are marked in red, nominally significant differences (TST) in black, and non-significant differences in grey.

### Sensitivity

Diagnostics showed that the regression models met key assumptions and yielded robust estimates. In the OLS models for log-transformed macro sleep outcomes (TST, SOL, WASO, REM latency, and sleep efficiency), RESET tests showed no evidence of misspecification for core models (*p*≥.28), with only REM latency showing some issues (*p*=.059), and all VIFs remained low (≤1.67). Influence diagnostics flagged at most four outlying residuals per model, and no Cook’s distances exceeded 0.04, indicating no single observation drove results (see Supplementary Table S3). In the quasi- and binomial generalized linear models for sleep staging (N1%, N2%, N3 presence/amount, REM presence/amount), RESET tests similarly showed no evidence of misspecification (all *p*≥.50), VIFs did not exceed 1.88, and dispersion parameters fell within acceptable ranges (ϕ ≈ 0.07–1.05). The N1% model did exhibit elevated influence and leverage (Cook’s distance = 0.07), though analyses excluding these cases (see Supplementary Table S4) yielded only a slightly reduced effect estimate and significance level (β = 0.189 [0.047‚ 0.329]; *p*=.009, q = 0.032). For the stage presence models for N3 and REM, HL tests confirmed good fit (*p*≥.24), and models demonstrated moderate explanatory power (pseudo-R^2^ = 0.14–0.18; ROC-AUC = 0.76–0.83) (see Supplementary Table S3).

Effect sizes were largely consistent across eight sensitivity models (see Supplementary Table S5A–D). An increase in WASO and reduction in sleep efficiency among chronic cannabis users was stable and reached nominal significance in six out of eight sensitivity analyses (WASO β = +0.126–190, *p*=.002–.032; sleep efficiency β = −0.122–0.160, *p*=.005–.025), with the exception of the most minimal specification (WASO β = +0.095, *p*=.105; sleep efficiency β = −0.102, *p*=.060) and the PSM-adjusted model, which showed similar estimates but failed to reached nominal significance (WASO β = +0.169, *p*=.055; sleep efficiency β = −0.139, *p*=.066). N1% showed a similar pattern, reaching nominal significance in six out of eight models (β = +0.199- + 0.254, *p* = < 0.001–0.005), with the exception of the ±3 *SD* trimmed (β = +0.130, *p*=.072) and the PSM-adjusted model (β = +0.160, *p*=.264). TST reached nominal significance in five out of eight analyses (β = −0.029, −0.034, *p*=.023–.047), with the exception of the ±3 *SD* trimmed (β = −0.028, *p*=.066), the most minimal (β = −0.027, *p*=.055), and the PSM-adjusted model (β = −0.020, *p*=.301). For N1, models holding TST, sleep efficiency, and WASO constant showed a similar estimate in the regular regression model (β = +0.173, *p*=.012) but not the PSM-adjusted model (β = +0.006, *p*=.958) (see Supplementary Table S6). In the PSM sample, the mean absolute standardized difference was 0.078, with all covariates ≤0.066 (see Supplementary Figure S1).

### Exploratory analyses

Across stratified models (Supplementary Table S7A–C), main effects—reduced TST and sleep efficiency, increased WASO and N1%—were directionally consistent across nearly all subgroups. Exceptions included N1% in the moderate apnea group (β = −0.003, *p*=.844, q = 0.935), and reversed trends among patients aged 65 and over, who showed increases in TST (β = +0.084, *p*=.068, q = 0.350) and sleep efficiency (β = +0.256, *p*=.117, q = 0.488), and a decrease in WASO (β = −0.151, *p*=.341, q = 0.720). However, none of these exceptions reached nominal significance. No interactions survived FDR correction (Supplementary Table S8). Three reached nominal significance: cannabis use predicted an increase in TST (β = 0.003, *p*=.014, q = 0.418) and sleep efficiency (β = 0.008, *p*=.043, q = 0.471) with age, and a lower likelihood of N3 sleep presence in men than in women (β = 1.425, *p*=.031, q = 0.470), though the latter had a relatively large standard error (SE = 0.662).

In sex-stratified models, none of the 11 outcomes reached nominal significance among women (all *p*≥.104). Among men, an FDR-significant reduction in sleep efficiency (β = −0.313, *p*=.001, q = 0.022), nominally significant increases in WASO (β = +0.286, *p*=.006, q = 0.051), N1% (β = +0.027, *p*=.007, q = 0.051), and SOL (β = +0.329, *p*=.028, q = 0.083), and a nominally significant decrease in TST (β = −0.049, *p*=.042, q = 0.100) were observed. SOL trended negatively for women (β = −0.166, *p*=.148, q = 0.326), though the cannabis x sex interaction for SOL did not reach nominal significance (β = 0.379, *p*=.068, q = 0.648).

No FDR-significant associations were found in any of the four groups in the stratified age analyses, though nominal significance was observed for N1% (β = +0.033, *p*=.011, q = 0.350) and N2% (β = −0.032, *p*=.037, q = 0.350) in the 18–34 group, and sleep efficiency (β = −0.201, *p*=.042, q = 0.350) in the 50–64 group.

In AHI-stratified models, no FDR-significant effects were observed in any AHI group. However, nominal significance was found for TST (β = −0.129, *p*=.006, q = 0.062) and sleep efficiency (β = −0.585, *p*=.004, q = 0.082) in the no-apnea group, for sleep efficiency (β = −0.317, *p*=.033, q = 0.271) and WASO (β = +0.428, *p*=.006, q = 0.082) in the moderate apnea group, and for N1% in the severe apnea group (β = +0.033, *p*=.021, q = 0.215).

Exploratory dose–response models showed no significant effect of duration or dose of cannabis use on any sleep outcome. Neither total years of use (*n* = 120), percentage of adult life using cannabis (*n* = 120), nor estimated dose—based on number of joints smoked (*n* = 45), grams used (*n* = 29), or STUs derived from these (*n* = 69)—were associated with macro- or sleep staging parameters. Estimated STUs ranged from 1.8 to 280 units (mean = 39.5; median = 20), years of use from 1 to 53 years (mean = 20; median = 17), and percent of adult life using cannabis from 2.3% to 100% (mean = 69.7%; median = 92.8%). Use patterns are presented in Supplementary Table S9 and dose–response analyses in Supplementary Table S10. Diagnostics supported stable inference for macro-sleep outcomes (TST, SOL, sleep efficiency, WASO, REM latency) but sleep-stage models remained unstable.

## Discussion

In this large, clinic-based study of patients referred primarily for suspected sleep apnea, chronic cannabis use was associated with more fragmented sleep: on average, wake after sleep onset (WASO) was 21 percent (~16.6 minutes) higher, sleep efficiency 3.8 percent lower, and N1% 2.8 percentage points higher compared to cannabis-naïve subjects. These effects persisted in directionality and magnitude across robustness checks and were not materially altered by the presence or severity of obstructive sleep apnea (OSA). A directional, though non-FDR-significant reduction in TST (3.3 percent; ~11.8 minutes) further suggests potential decrements in sleep quality.

Although the absolute magnitudes of these differences may appear modest, changes of this size may be considered clinically meaningful within sleep medicine. In healthy adults, self-reported WASO exceeding 30 minutes and sleep efficiency below 85 percent are generally regarded as indicative of clinically significant fragmentation [89, 90]. A 21 percent increase in WASO or a 3.8 percentage-point reduction in efficiency can shift patients from a near-normal to a pathological range, particularly in sleep-clinic populations who already exhibit reduced baseline efficiency.

Reduced sleep efficiency, driven by increased WASO, indicates fragmentation of the sleep period; a hallmark of poor sleep quality and predictive of daytime fatigue [91], cognitive impairment [92], and mood dysregulation [93, 94]. Small reductions in TST, when sustained over time, may contribute to chronic sleep insufficiency. A modest elevation in the proportion of N1, from a baseline already at the higher end of normative ranges, similarly suggests lighter, less stable sleep and a modest reduction in restorative potential [95]. Although other stage percentages did not reach statistical significance, all shifted directionally downward, consistent with a light compositional redistribution of non-REM sleep toward lighter stages. This elevation in N1 persisted when macro-sleep variables were held constant.

These alterations in sleep architecture may have broader functional implications. Chronic cannabis use has been associated with attentional deficits [96], impaired executive functioning [97, 98], and mood dysregulation [99, 100], all of which are domains sensitive to disrupted sleep continuity [94, 101–104]. The elevated N1 proportion and lower efficiency observed here may therefore contribute to daytime cognitive and affective symptoms frequently reported in chronic users.

This points to a broader public health concern: cannabis is widely used as a sleep aid, particularly among high school [105] and college students [106], yet chronic use may worsen sleep difficulties. Tolerance to cannabis’ sedating effects and withdrawal-related sleep disturbances [107–110] may contribute to a cycle of self-medication and further sleep disruption [43], potentially exacerbating rather than resolving sleep difficulties.

That said, the directionality of effects remains uncertain. It is plausible that individuals with more severe sleeping difficulties are more likely to use cannabis in an attempt to manage their symptoms, which may explain their poorer sleep architecture. Although the characteristics of cannabis users in this study generally align with those reported in epidemiological research [41, 111–116], significant differences in cardiovascular conditions, even after adjusting for age and sex, suggest residual confounding. Individuals using cannabis in the study may have differed in ways from non-users that could not be adequately controlled for.

Further, within-group dose–response analyses did not indicate that longer use duration or higher estimated THC exposure was associated with more impaired sleep. However, these analyses were limited by small sample sizes and relatively unreliable exposure estimates. In response to growing calls for cannabis research to incorporate measures of dose and frequency, our study illustrates a fundamental practical challenge: cannabis products and methods vary widely in (absorbed) THC [52, 53], and accurately inferring the amount of THC consumed from patients’ self-report—mixing vaping, smoking, and edible use, each with distinct THC:CBD ratios, potencies, and doses—is highly complex. Joints vary substantially by weight and grams of cannabis by potency, and users commonly overestimate the number of grams in their products [117], rendering these self-report inferences unreliable indicators of actual THC exposure for dose–response analyses.

Our findings diverge in several respects from those of existing studies, which have typically reported null or inconsistent effects of cannabis on sleep architecture. Most clinical trials find no significant impact on WASO or sleep efficiency when administering cannabis to patients [14, 19, 20, 22, 25, 26, 28, 79–82], although withdrawal from the substance has been associated with increased WASO and reduced sleep efficiency [108, 118, 119]. TST also tends to remain unchanged in the clinical cannabis literature [15, 21, 23, 107, 108, 118–122]. Similarly, elevated N1 sleep does not reflect previous trial findings, which have only reported reduced [122, 123] or unchanged N1 [14, 22, 28, 82]. However, reflecting the findings of the present study, a recent cross-sectional study using home sleep apnea testing reported increases in WASO and N1 among individuals who regularly use cannabis [124].

Further contrasting our findings with early clinical trials [107, 108, 120, 122, 123], but in line with more recent trials using lower THC doses [15, 21, 23, 121] and observational studies [36–38], REM sleep did not show significant impairment in this study. Although the proportionally higher share of N1 indicates less time spent in other sleep stages, the data did not indicate that REM sleep was disproportionately affected compared to other stages. This may reflect the homeostatic resilience of REM sleep [125], which tends to rebound and normalize across nights [107, 108, 120].

The discrepancies in findings between our study and existing trials may partly reflect the limited statistical power of such trials, which typically include fewer than 30 participants and administer relatively low doses of cannabis [15, 20, 23, 121, 126]. Given the high inter-individual variability inherent in sleep architecture measures, these studies may be underpowered to detect modest effects. By leveraging a larger clinical sample, our study offers increased sensitivity to detect small alterations. Further, clinical trials standardize timing, dosage, and administration route, deviating from the most common use method—inhalation [9, 10, 27]—whereas our observational data reflect naturalistic, long-term patterns of use while controlling for key demographic, clinical, and pharmacologic covariates. Indeed, 95 percent of our sample indicated they inhaled cannabis, with only 5 percent using edibles as their primary method.

To our knowledge, this is the first study to examine chronic cannabis use and sleep architecture in a relatively large sample of cannabis users assessed with in-laboratory PSG. Our sample appears to reflect real-world cannabis use patterns both in terms of prevalence and profile: 10.4 percent of patients met criteria for chronic use, aligning with national estimates in Canada [41], and, consistent with broader epidemiological data, cannabis users in our sample were more likely to be male [41], younger [85], and reported higher rates of nicotine use [112], psychiatric diagnoses [113], and use of sedatives [114], antidepressants [115], antipsychotics [115], and opioids [116]—patterns well-established in the cannabis literature.

However, the study’s external validity is simultaneously strongly limited by the fact that it was conducted at a sleep clinic, as the clinic largely performs studies for the detection of OSA—only 9.6 percent of the overall sample did not have apnea, and 44 percent had clinically severe OSA. Patients showed an average sleep duration of just 6 hours, far below the “normal” range of 7 to 9 hours, as well as poor sleep efficiencies, prolonged WASO [127–129], significantly prolonged REM latencies [130], elevated N1 sleep, and significantly reduced N3 and REM sleep [131]. This suggests caution when generalizing to healthy sleepers, as findings may reflect cannabis and pre-existing sleep pathology interactions. However, this limitation is somewhat tempered by the study’s internal validity and analytic controls. Further, stratifying by AHI level did not alter the directionality of findings.

Several other significant limitations need to be acknowledged. Our study’s dose–response analyses were based on relatively broad assumptions. Stronger causal inferences could be made if precise cannabis type, formulation, dose, use frequency, and potency were collected to estimate overall THC and CBD intake; future research would strongly benefit from finding ways to more accurately measure this. Further, caffeine use, a known disruptor of sleep architecture [17, 132], was not controlled for. Although the clinic strictly instructs patients to avoid caffeine after 2PM on the day of their study, verifying compliance is challenging and reliable self-report beyond this point is difficult to obtain. However, there is no indication that any such violations would differ systematically between cannabis users and non-users. In addition, not every patient may have honestly disclosed their cannabis use, potentially due to stigma [133], though the prevalence and characteristics of the cannabis using sample align well with epidemiological trends in chronic cannabis use. Psychiatric conditions could also not be meaningfully split between depression and anxiety, which have important neuro- and pathophysiological differences. The cross-sectional design of the study further precludes establishing temporal order and residual confounding remains an issue.

A final notable limitation involves the role of prescription drug use. In the main model, medications were included as covariates to control for pharmacological influences on sleep. However, this assumes equivalent patterns of use across groups, which may not hold. Daily cannabis users often engage in substitution, using cannabis in place of prescription sleep aids, opioids, or anxiolytics [134, 135], while non-users may rely more heavily on such drugs—for example, in our study, the share of patients with psychiatric disorders that used antidepressants was significantly smaller among those who used cannabis than those who did not. As a result, controlling for medications could partially adjust away the differences under investigation. However, sensitivity models excluding medication covariates, morbidity covariates, or both, produced similar results: cannabis use remained associated with increased WASO, reduced sleep efficiency, and modest reductions in total sleep time and elevated N1 sleep. This consistency suggests that substitution effects, while important to consider, do not fully account for the observed associations.

These patterns highlight the need for future research to move beyond binary definitions of cannabis exposure toward dose-responsive models that capture THC:CBD ratios, route of administration, nightly timing, and proximity to sleep onset, where prospective cohort designs and randomized controlled studies could clarify whether sleep disruptions vary according to cannabis formulations and use patterns. Given the indication of greater sleep disruption among younger users, adolescent and emerging adult populations warrant particular attention. Finally, examining whether low-dose therapeutic cannabis similarly impairs sleep continuity remains an important question, particularly to guide safer clinical recommendations and informing public health guidelines.

## Supplementary Material

Figure_S1_Love_plot_zsaf396

SLEEP_Supplementary_Materials_for_publication_zsaf396

## Data Availability

The data underlying this article cannot be shared publicly due to privacy restrictions from the clinical sleep center. De-identified data may be shared upon reasonable request to the corresponding author.

## References

[1] Leung J, Chan G, Stjepanović D, Chung JYC, Hall W, Hammond D. Prevalence and self-reported reasons of cannabis use for medical purposes in USA and Canada. *Psychopharmacology (Berl)*. 2022;239(5):1509–1519. 10.1007/s00213-021-06047-8 35020045 PMC9110511

[2] Goodman S, Wadsworth E, Leos-Toro C, Hammond D. Prevalence and forms of cannabis use in legal vs. illegal recreational cannabis markets. *Int J Drug Policy*. 2020;76:102658. 10.1016/j.drugpo.2019.102658 31927413

[3] Walsh Z, Callaway R, Belle-Isle L, et al. Cannabis for therapeutic purposes: patient characteristics, access, and reasons for use. *Int J Drug Policy*. 2013;24(6):511–516. 10.1016/j.drugpo.2013.08.010 24095000

[4] Turna J, Balodis I, Munn C, Van Ameringen M, Busse J, MacKillop J. Overlapping patterns of recreational and medical cannabis use in a large community sample of cannabis users. *Compr Psychiatry*. 2020;102:152188. 10.1016/j.comppsych.2020.152188 32653594

[5] Chandy M, Nishiga M, Wei TT, Hamburg NM, Nadeau K, Wu JC. Adverse impact of cannabis on human health. *Annu Rev Med*. 2024;75:353–367. 10.1146/annurev-med-052422-020627 37582489 PMC10947506

[6] Gorelick DA . Cannabis-related disorders and toxic effects. *N Engl J Med*. 2023;389(24):2267–2275. 10.1056/NEJMra2212152 38091532

[7] Lorenzetti V, Hoch E, Hall W. Adolescent cannabis use, cognition, brain health and educational outcomes: a review of the evidence. *Eur Neuropsychopharmacol*. 2020;36:169–180. 10.1016/j.euroneuro.2020.03.012 32268974

[8] Walker MP . The role of sleep in cognition and emotion. *Ann N Y Acad Sci*. 2009;1156(1):168–197. 10.1111/j.1749-6632.2009.04416.x 19338508

[9] Velzeboer R, Malas A, Boerkoel P, et al. Cannabis dosing and administration for sleep: a systematic review. *Sleep.* 2022;45(11). 10.1093/sleep/zsac218 36107800

[10] AminiLari M, Wang L, Neumark S, et al. Medical cannabis and cannabinoids for impaired sleep: a systematic review and meta-analysis of randomized clinical trials. *Sleep.* 2022;45(2). 10.1093/sleep/zsab234 34546363

[11] Rundo JV, Downey R. Chapter 25—polysomnography. In: Levin KH, Chauvel P, eds. Handbook of Clinical Neurology. Vol 160. Clinical Neurophysiology: Basis and Technical Aspects. Amsterdam, Netherlands: Elsevier; 2019:381–392. 10.1016/B978-0-444-64032-1.00025-4 31277862

[12] Edwards D, Filbey FM. Are sweet dreams made of these? Understanding the relationship between sleep and cannabis use. *Cannabis Cannabinoid Res*. 2021;6(6):462–473. 10.1089/can.2020.0174 34143657 PMC8713269

[13] Velzeboer R, Malas A, Wei S, Berger R, Parmar V, Lai WWK. Cannabis and sleep architecture: a systematic review and meta-analysis. *Sleep Med Rev*. 2025;84:102164. 10.1016/j.smrv.2025.102164 40967124

[14] Sznitman SR, Shochat T, Greene T. Is time elapsed between cannabis use and sleep start time associated with sleep continuity? An experience sampling method. *Drug Alcohol Depend*. 2020;208:107846. 10.1016/j.drugalcdep.2020.107846 31954953

[15] Nicholson AN, Turner C, Stone BM, Robson PJ. Effect of Δ-9-tetrahydrocannabinol and cannabidiol on nocturnal sleep and early-morning behavior in young adults. *J Clin Psychopharmacol*. 2004;24(3):305. 10.1097/01.jcp.0000125688.05091.8f 15118485

[16] Babson KA, Sottile J, Morabito D. Cannabis, cannabinoids, and sleep: a review of the literature. *Curr Psychiatry Rep*. 2017;19(4):23. 10.1007/s11920-017-0775-9 28349316

[17] Garcia AN, Salloum IM. Polysomnographic sleep disturbances in nicotine, caffeine, alcohol, cocaine, opioid, and cannabis use: a focused review. *Am J Addict*. 2015;24(7):590–598. 10.1111/ajad.12291 26346395

[18] Scullin MK, Gao C. Dynamic contributions of slow wave sleep and REM sleep to cognitive longevity. *Curr Sleep Med Rep*. 2018;4(4):284–293. 10.1007/s40675-018-0131-6 31737466 PMC6857934

[19] Della Monica C, Johnsen S, Atzori G, Groeger JA, Dijk DJ. Rapid eye movement sleep, sleep continuity and slow wave sleep as predictors of cognition, mood, and subjective sleep quality in healthy men and women, aged 20–84 years. *Front Psych*. 2018;9:255. 10.3389/fpsyt.2018.00255 PMC6024010 29988413

[20] Linares IMP, Guimaraes FS, Eckeli A, et al. No acute effects of cannabidiol on the sleep-wake cycle of healthy subjects: a randomized, double-blind, placebo-controlled, crossover study. *Front Pharmacol*. 2018;9:315. 10.3389/fphar.2018.00315 PMC5895650 29674967

[21] Carley DW, Prasad B, Reid KJ, et al. Pharmacotherapy of apnea by cannabimimetic enhancement, the PACE clinical trial: effects of dronabinol in obstructive sleep apnea. *Sleep.* 2018;41(1). 10.1093/sleep/zsx184 PMC5806568 29121334

[22] Prasad B, Radulovacki MG, Carley DW. Proof of concept trial of dronabinol in obstructive sleep apnea. *Front Psych*. 2013;4:1. 10.3389/fpsyt.2013.00001 PMC3550518 23346060

[23] Farabi SS, Prasad B, Quinn L, Carley DW. Impact of donabinol on quantitative electroencephalogram (qEEG) measures of sleep in obstructive sleep apnea syndrome. *J Clin Sleep Med*. 2014;10(01):49–56. 10.5664/jcsm.3358 24426820 PMC3869068

[24] Bruni N, Della Pepa C, Oliaro-Bosso S, Pessione E, Gastaldi D, Dosio F. Cannabinoid delivery systems for pain and inflammation treatment. *Molecules.* 2018;23(10):2478. 10.3390/molecules23102478 30262735 PMC6222489

[25] Ware MA, Wang T, Shapiro S, et al. Smoked cannabis for chronic neuropathic pain: a randomized controlled trial. *Can Med Assoc J*. 2010;182(14):E694–E701. 10.1503/cmaj.091414 20805210 PMC2950205

[26] Bidwell LC, Karoly HC, Torres MO, Master A, Bryan AD, Hutchison KE. A naturalistic study of orally administered vs. inhaled legal market cannabis: cannabinoids exposure, intoxication, and impairment. *Psychopharmacology (Berl)*. 2022;239(2):385–397. 10.1007/s00213-021-06007-2 34708254 PMC12238766

[27] Schauer GL, Njai R, Grant-Lenzy AM. Modes of marijuana use—smoking, vaping, eating, and dabbing: results from the 2016 BRFSS in 12 states. *Drug Alcohol Depend*. 2020;209:107900. 10.1016/j.drugalcdep.2020.107900 32061947 PMC11734406

[28] Atakan Z . Cannabis, a complex plant: different compounds and different effects on individuals. *Ther Adv Psychopharmacol*. 2012;2(6):241–254. 10.1177/2045125312457586 23983983 PMC3736954

[29] da Silva GHS, Barbosa EC, de Lima FR, et al. Effectiveness of cannabinoids on subjective sleep quality in people with and without insomnia or poor sleep: a systematic review and meta-analysis of randomised studies. *Sleep Med Rev*. 2025;84:102156. 10.1016/j.smrv.2025.102156 40929927

[30] Ranum RM, Whipple MO, Croghan I, Bauer B, Toussaint LL, Vincent A. Use of cannabidiol in the management of insomnia: a systematic review. *Cannabis Cannabinoid Res*. 2023;8(2):213–229. 10.1089/can.2022.0122 36149724

[31] Lavender I, Garden G, Grunstein RR, Yee BJ, Hoyos CM. Using cannabis and CBD to sleep: an updated review. *Curr Psychiatry Rep*. 2024;26(12):712–727. 10.1007/s11920-024-01564-7 39612156

[32] Bonn-Miller MO, Feldner MT, Bynion TM, et al. A double-blind, randomized, placebo-controlled study of the safety and effects of CBN with and without CBD on sleep quality. *Exp Clin Psychopharmacol*. 2024;32(3):277–284. 10.1037/pha0000682 37796540

[33] Kolobaric A, Saleska J, Hewlings SJ, et al. A randomized, double-blind, placebo-controlled trial to assess the effectiveness and safety of melatonin and three formulations of Floraworks proprietary TruCBN™ for improving sleep. *Pharmaceuticals.* 2024;17(8):977. 10.3390/ph17080977 PMC11357382 39204082

[34] Hall W, Degenhardt L. The adverse health effects of chronic cannabis use. *Drug Test Anal*. 2014;6(1-2):39–45. 10.1002/dta.1506 23836598

[35] Gates P, Albertella L, Copeland J. Cannabis withdrawal and sleep: a systematic review of human studies. *Subst Abuse*. 2016;37(1):255–269. 10.1080/08897077.2015.1023484 25893849

[36] Pranikoff K, Karacan I, Larson EA, Williams RL, Thornby JI, Hursch CJ. Effects of marijuana smoking on the sleep EEG. Preliminary studies. *JFMA J Fla Med Assoc Fla Med Assoc*. 1973;60(3):28–31 4347054

[37] Karacan I, Fernández-Salas A, Coggins WJ, et al. Sleep electroencephalographic-electrooculographic characteristics of chronic marijuana users: part I. *Ann N Y Acad Sci*. 1976;282(1):348–374. 10.1111/j.1749-6632.1976.tb49909.x 190937

[38] Bolla KI, Lesage SR, Gamaldo CE, et al. Sleep disturbance in heavy marijuana users. *Sleep.* 2008;31(6):901–908. 10.1093/sleep/31.6.901 18548836 PMC2442418

[39] Whitehurst LN, Fogler K, Hall K, Hartmann M, Dyche J. The effects of chronic marijuana use on circadian entrainment. *Chronobiol Int*. 2015;32(4):561–567. 10.3109/07420528.2015.1004078 25801606

[40] Veitch MR, Jairam S, Gurges P, et al. Cannabinoid use and obstructive sleep apnea: a retrospective cohort study. *Can J Neurol Sci*. 2024;22:1–8. 10.1017/cjn.2024.25 38383993

[41] Hammond D, Goodman S, Wadsworth E, et al. Trends in the use of cannabis products in Canada and the USA, 2018–2020: findings from the International Cannabis Policy Study. *Int J Drug Policy*. 2022;105:103716. 10.1016/j.drugpo.2022.103716 35613480

[42] Sancho-Domingo C, Carballo JL, Coloma-Carmona A, Pelegrín Muñoz A, van-der Hofstadt C. Alcohol and cannabis as sleep aids among adolescents and associations with sleep quality and problematic use. *Addict Behav*. 2025;165:108304. 10.1016/j.addbeh.2025.108304 39999517

[43] Goodhines PA, Rathod K, Cingranelli L. Sleep health, self-medication, and cannabis risk: a bidirectional model and research agenda. *Curr Sleep Med Rep*. 2025;11(1):4. 10.1007/s40675-024-00314-8

[44] Costa NL, Silva WCDSE, Silva MLD, Cunha KDC. Sleep quality of adult recreational cannabis users: a systematic literature review. *Trends Psychiatry Psychother*. 2025;47:e20240802. 10.47626/2237-6089-2024-0802 38754072 PMC12908920

[45] Shrivastava D, Syung J, Mohsen S, Roopa S, Crewson K. How to interpret the results of a sleep study. *J Community Hosp Intern Med Perspect*. 2014;4(5):24983. 10.3402/jchimp.v4.24983 25432643 PMC4246141

[46] Kapur VK, Auckley DH, Chowdhuri S, et al. Clinical practice guideline for diagnostic testing for adult obstructive sleep apnea: an American Academy of Sleep Medicine Clinical Practice Guideline. *J Clin Sleep Med*. 13(3):479–504. 10.5664/jcsm.6506 28162150 PMC5337595

[47] Jones RT, Benowitz NL, Herning RI. Clinical relevance of cannabis tolerance and dependence. *J Clin Pharmacol*. 1981;21(S1):143S–152S. 10.1002/j.1552-4604.1981.tb02589.x 6271820

[48] Theunissen EL, Kauert GF, Toennes SW, et al. Neurophysiological functioning of occasional and heavy cannabis users during THC intoxication. *Psychopharmacology (Berl)*. 2012;220(2):341–350. 10.1007/s00213-011-2479-x 21975580 PMC3285765

[49] Fridberg DJ, Skosnik PD, Hetrick WP, O’Donnell BF. Neural correlates of performance monitoring in chronic cannabis users and cannabis-naïve controls. *J Psychopharmacol*. 2013;27(6):515–525. 10.1177/0269881113477745 23427191 PMC3923357

[50] Sun R, Mendez D, Warner KE. Use of electronic cigarettes among cannabis-naive adolescents and its association with future cannabis use. *JAMA Netw Open*. 2022;5(7):e2223277. 10.1001/jamanetworkopen.2022.23277 35867059 PMC9308048

[51] Sexton M, Silvestroni A, Möller T, Stella N. Differential migratory properties of monocytes isolated from human subjects naïve and non-naïve to cannabis. *Inflammopharmacology.* 2013;21(3):253–259. 10.1007/s10787-012-0133-9 22492174 PMC4476512

[52] Chayasirisobhon S . Mechanisms of action and pharmacokinetics of cannabis. *Perm J*. 2020;25:1–3. 10.7812/TPP/19.200 PMC8803256 33635755

[53] Wood S, Gabrys R, Freeman T, Hammond D. Canada’s THC unit: applications for the legal cannabis market. *Int J Drug Policy*. 2024;128:104457. 10.1016/j.drugpo.2024.104457 38772194

[54] Borodovsky JT, Hasin DS, Shmulewitz D, et al. Typical hits, grams, or joints: evaluating cannabis survey measurement strategies for quantifying consumption. *Cannabis Cannabinoid Res.* 2024;9(2):646–658. 10.1089/can.2022.0237 36577020 PMC10998027

[55] Cannabis in British Columbia: Results from the 2021 BC Cannabis Use Survey. Vol. 35. Victoria, BC, Canada: BC Stats; 2022.

[56] Tassone F, Di Ciano P, Liu Y, Rueda S. On offer to Ontario consumers three years after legalization: a profile of cannabis products, cannabinoid content, plant type, and prices. *Front Psych*. 2023;14:1111330. 10.3389/fpsyt.2023.1111330 PMC9978145 36873222

[57] Freeman TP, Lorenzetti V. A standard THC unit for reporting of health research on cannabis and cannabinoids. *Lancet Psychiatry*. 2021;8(11):944–946. 10.1016/S2215-0366(21)00355-2 34506750

[58] Freeman TP, Lorenzetti V. ‘Standard THC units’: a proposal to standardize dose across all cannabis products and methods of administration. *Addiction.* 2020;115(7):1207–1216. 10.1111/add.14842 31606008

[59] Baker FC, Yűksel D, de Zambotti M. Sex differences in sleep. In: Attarian H, Viola-Saltzman M, eds. Sleep Disorders in Women: A Guide to Practical Management. Cham, Switzerland: Springer International Publishing; 2020:55–64. 10.1007/978-3-030-40842-8_5

[60] Dorffner G, Vitr M, Anderer P. The effects of aging on sleep architecture in healthy subjects. In: Vlamos P, Alexiou A, eds. GeNeDis 2014. Cham, Switzerland: Springer International Publishing; 2015:93–100. 10.1007/978-3-319-08939-3_13 25416113

[61] Moraes W, Poyares D, Zalcman I, et al. Association between body mass index and sleep duration assessed by objective methods in a representative sample of the adult population. *Sleep Med*. 2013;14(4):312–318. 10.1016/j.sleep.2012.11.010 23391395

[62] Rao MN, Blackwell T, Redline S, et al. Association between sleep architecture and measures of body composition. *Sleep.* 2009;32(4):483–490. 10.5665/sleep/32.4.483 19413142 PMC2663862

[63] Chang WP, Peng YX. Meta-analysis of differences in sleep quality based on actigraphs between day and night shift workers and the moderating effect of age. *J Occup Health*. 2021;63(1):e12262. 10.1002/1348-9585.12262 34392580 PMC8364763

[64] Yang P, Weng J, Huang X. Sleep features in alcohol use disorder: a systematic review and meta-analysis of polysomnographic findings in case-control studies. *Eur J Psychiatry*. 2024;38(2):100231. 10.1016/j.ejpsy.2023.100231

[65] Kanclerska J, Szymańska-Chabowska A, Poręba R, et al. A systematic review of publications on the associations between sleep architecture and arterial hypertension. *Med Sci Monit*. 2023;29:e941066. 10.12659/MSM.941066 37665688 PMC10487188

[66] Shekhar S, Hall JE, Klubo-Gwiezdzinska J. The hypothalamic–pituitary–thyroid axis and sleep. *Curr Opin Endocr Metab Res*. 2021;17:8–14. 10.1016/j.coemr.2020.10.002 34322645 PMC8315115

[67] Chen DM, Taporoski TP, Alexandria SJ, et al. Altered sleep architecture in diabetes and prediabetes: findings from the Baependi Heart Study. *Sleep.* 2024;47(1). 10.1093/sleep/zsad229 PMC13070545 37658822

[68] Khanijow V, Prakash P, Emsellem HA, Borum ML, Doman DB. Sleep dysfunction and gastrointestinal diseases. *Gastroenterol Hepatol*. 2015;11(12):817–825 PMC4849511 27134599

[69] Stefani A, Högl B. Sleep in Parkinson’s disease. *Neuropsychopharmacology.* 2020;45(1):121–128. 10.1038/s41386-019-0448-y 31234200 PMC6879568

[70] Harding SM . Sleep in fibromyalgia patients: subjective and objective findings. *Am J Med Sci*. 1998;315(6):367–376. 10.1016/S0002-9629(15)40354-4 9638893

[71] Onen SH, Onen F, Courpron P, Dubray C. How pain and analgesics disturb sleep. *Clin J Pain*. 2005;21(5):422. 10.1097/01.ajp.0000129757.31856.f7 16093748

[72] Pandi-Perumal SR, Monti JM, Burman D, et al. Clarifying the role of sleep in depression: a narrative review. *Psychiatry Res*. 2020;291:113239. 10.1016/j.psychres.2020.113239 32593854

[73] Papadimitriou GN, Linkowski P. Sleep disturbance in anxiety disorders. *Int Rev Psychiatry*. 2005;17(4):229–236. 10.1080/09540260500104524 16194794

[74] Rao R, Tripathi R. Stimulants and sleep. In: Gupta R, Neubauer DN, Pandi-Perumal SR, eds. Sleep and Neuropsychiatric Disorders. Cham, Switzerland: Springer Nature; 2022:811–833. 10.1007/978-981-16-0123-1_40

[75] McEntire DM, Kirkpatrick DR, Kerfeld MJ, et al. Effect of sedative-hypnotics, anesthetics and analgesics on sleep architecture in obstructive sleep apnea. *Expert Rev Clin Pharmacol*. 2014;7(6):787–806. 10.1586/17512433.2014.966815 25318836

[76] Doghramji K, Jangro WC. Adverse effects of psychotropic medications on sleep. *Sleep Med Clin*. 2016;11(4):503–514. 10.1016/j.jsmc.2016.08.001 28118873

[77] Ghossoub E, Geagea L, Kobeissy F, Talih F. Comparative effects of psychotropic medications on sleep architecture: a retrospective review of diagnostic polysomnography sleep parameters. *Sleep Sci*. 2021;14(3):236–244. 10.5935/1984-0063.20200071 35186202 PMC8848521

[78] Ongini E, Milani S, Marzanatti M, Trampus M, Monopoli A. Effects of selected beta-adrenergic blocking agents on sleep stages in spontaneously hypertensive rats. *J Pharmacol Exp Ther*. 1991;257(1):114–119. 10.1016/S0022-3565(25)24654-7 1673471

[79] Fehm HL, Benkowitsch R, Kern W, Fehm-Wolfsdorf G, Pauschinger P, Born J. Influences of corticosteroids, dexamethasone and hydrocortisone on sleep in humans. *Neuropsychobiology.* 2008;16(4):198–204. 10.1159/000118326 3614616

[80] Oelke M, De Wachter S, Drake MJ, et al. A practical approach to the management of nocturia. *Int J Clin Pract*. 2017;71(11):e13027. 10.1111/ijcp.13027 28984060 PMC5698733

[81] Mann C, Wegner J, Weeß HG, Staubach P. Pathobiology of second-generation antihistamines related to sleep in urticaria patients. *Biology.* 2022;11(3):433. 10.3390/biology11030433 35336805 PMC8945773

[82] Scanga A, Lafontaine AL, Kaminska M. An overview of the effects of levodopa and dopaminergic agonists on sleep disorders in Parkinson’s disease. *J Clin Sleep Med*. 19(6):1133–1144. 10.5664/jcsm.10450 PMC10235717 36716191

[83] Dimsdale JE, Norman D, DeJardin D, Wallace MS. The effect of opioids on sleep architecture. *J Clin Sleep Med*. 2007;3(1):33–36 17557450

[84] McArdle N, Douglas NJ. Effect of continuous positive airway pressure on sleep architecture in the sleep apnea-hypopnea syndrome: a randomized controlled trial. *Am J Respir Crit Care Med*. 2001;164(8 Pt 1):1459–1463. 10.1164/ajrccm.164.8.2008146 11704596

[85] Espa F, Ondze B, Deglise P, Billiard M, Besset A. Sleep architecture, slow wave activity, and sleep spindles in adult patients with sleepwalking and sleep terrors. *Clin Neurophysiol*. 2000;111(5):929–939. 10.1016/S1388-2457(00)00249-2 10802466

[86] Mancebo-Sosa V, Mancilla-Hernández V, Miranda-Ortiz J, et al. Sleep architecture alterations in patients with periodic limb movements disorder during sleep and sleep breathing disorders. *Sleep Sci*. 2016;9(2):84–88. 10.1016/j.slsci.2016.06.002 27656271 PMC5022004

[87] R Core Team . R: A Language and Environment for Statistical Computing. 2024. Published online. https://www.R-project.org/

[88] de Boer MR, Waterlander WE, Kuijper LD, Steenhuis IH, Twisk JW. Testing for baseline differences in randomized controlled trials: an unhealthy research behavior that is hard to eradicate. *Int J Behav Nutr Phys Act*. 2015;12:4. 10.1186/s12966-015-0162-z 25616598 PMC4310023

[89] Lichstein KL, Durrence HH, Taylor DJ, Bush AJ, Riedel BW. Quantitative criteria for insomnia. *Behav Res Ther*. 2003;41(4):427–445. 10.1016/S0005-7967(02)00023-2 12643966

[90] Reed DL, Sacco WP. Measuring sleep efficiency: what should the denominator be? *J Clin Sleep Med*. 2016;12(2):263–266. 10.5664/jcsm.5498 26194727 PMC4751425

[91] Dawson D, McCulloch K. Managing fatigue: it’s about sleep. *Sleep Med Rev*. 2005;9(5):365–380. 10.1016/j.smrv.2005.03.002 16099184

[92] Blackwell T, Yaffe K, Ancoli-Israel S, et al. Association of sleep characteristics and cognition in older community-dwelling men: the MrOS sleep study. *Sleep.* 2011;34(10):1347–1356. 10.5665/SLEEP.1276 21966066 PMC3174836

[93] Baum KT, Desai A, Field J, Miller LE, Rausch J, Beebe DW. Sleep restriction worsens mood and emotion regulation in adolescents. *J Child Psychol Psychiatry*. 2014;55(2):180–190. 10.1111/jcpp.12125 24889207 PMC4047523

[94] Konjarski M, Murray G, Lee VV, Jackson ML. Reciprocal relationships between daily sleep and mood: a systematic review of naturalistic prospective studies. *Sleep Med Rev*. 2018;42:47–58. 10.1016/j.smrv.2018.05.005 30404728

[95] Walker MP, van der Helm E. Overnight therapy? The role of sleep in emotional brain processing. *Psychol Bull*. 2009;135(5):731–748. 10.1037/a0016570 19702380 PMC2890316

[96] Lundqvist T . Cognitive consequences of cannabis use: comparison with abuse of stimulants and heroin with regard to attention, memory and executive functions. *Pharmacol Biochem Behav*. 2005;81(2):319–330. 10.1016/j.pbb.2005.02.017 15925403

[97] Crean RD, Crane NA, Mason BJ. An evidence-based review of acute and long-term effects of cannabis use on executive cognitive functions. *J Addict Med*. 2011;5(1):1. 10.1097/ADM.0b013e31820c23fa 21321675 PMC3037578

[98] Cohen K, Weinstein A. The effects of cannabinoids on executive functions: evidence from cannabis and synthetic cannabinoids—a systematic review. *Brain Sci*. 2018;8(3):40. 10.3390/brainsci8030040 PMC5870358 29495540

[99] Sorkhou M, Dent EL, George TP. Cannabis use and mood disorders: a systematic review. *Front Public Health*. 2024;12:1346207. 10.3389/fpubh.2024.1346207 PMC11035759 38655516

[100] Kuhns L, Kroon E, Colyer-Patel K, Cousijn J. Associations between cannabis use, cannabis use disorder, and mood disorders: longitudinal, genetic, and neurocognitive evidence. *Psychopharmacology (Berl)*. 2022;239(5):1231–1249. 10.1007/s00213-021-06001-8 34741634 PMC9520129

[101] Hudson AN, Van Dongen HPA, Honn KA. Sleep deprivation, vigilant attention, and brain function: a review. *Neuropsychopharmacology.* 2020;45(1):21–30. 10.1038/s41386-019-0432-6 31176308 PMC6879580

[102] Rodrigues T, Shigaeff N. Sleep disorders and attention: a systematic review. *Arq Neuropsiquiatr*. 2022;80(5):530–538. 10.1590/0004-282X-ANP-2021-0182 35476076 PMC9238330

[103] Wilckens KA, Woo SG, Kirk AR, Erickson KI, Wheeler ME. Role of sleep continuity and total sleep time in executive function across the adult lifespan. *Psychol Aging*. 2014;29(3):658–665. 10.1037/a0037234 25244484 PMC4369772

[104] Lowe CJ, Safati A, Hall PA. The neurocognitive consequences of sleep restriction: a meta-analytic review. *Neurosci Biobehav Rev*. 2017;80:586–604. 10.1016/j.neubiorev.2017.07.010 28757454

[105] Goodhines PA, Wedel AV, Dobani F, Zaso MJ, Gellis LA, Park A. Cannabis use for sleep aid among high school students: concurrent and prospective associations with substance use and sleep problems. *Addict Behav*. 2022;134:107427. 10.1016/j.addbeh.2022.107427 35872526 PMC9999445

[106] Graupensperger S, Hultgren BA, Fairlie AM, Lee CM, Larimer ME. Using alcohol and cannabis as sleep aids: associations with descriptive norms among college students. *Behav Sleep Med*. 2023;21(1):84–96. 10.1080/15402002.2022.2040505 35156478 PMC9372229

[107] Feinberg I, Jones R, Walker J, Cavness C, Floyd T. Effects of marijuana extract and tetrahydrocannabinol on electroencephalographic sleep patterns. *Clin Pharmacol Ther*. 1976;19(6):782–794. 10.1002/cpt1976196782 178475

[108] Feinberg I, Jones R, Walker JM, Cavness C, March J. Effects of high dosage delta-9-tetrahydrocannabinol on sleep patterns in man. *Clin Pharmacol Ther*. 1975;17(4):458–466. 10.1002/cpt1975174458 164314

[109] Vandrey R, Smith MT, McCann UD, Budney AJ, Curran EM. Sleep disturbance and the effects of extended-release zolpidem during cannabis withdrawal. *Drug Alcohol Depend*. 2011;117(1):38–44. 10.1016/j.drugalcdep.2011.01.003 21296508 PMC3119729

[110] Bolla KI, Lesage SR, Gamaldo CE, et al. Polysomnogram changes in marijuana users who report sleep disturbances during prior abstinence. *Sleep Med*. 2010;11(9):882–889. 10.1016/j.sleep.2010.02.013 20685163 PMC2938870

[111] Health Canada . Canadian Cannabis Survey 2024: Summary. Ottawa, ON, Canada: Health Canada; 2024. Accessed April 21, 2025. https://www.canada.ca/en/health-canada/services/drugs-medication/cannabis/research-data/canadian-cannabis-survey-2024-summary.html

[112] Goodwin RD, Pacek LR, Copeland J, et al. Trends in daily cannabis use among cigarette smokers: United States, 2002–2014. *Am J Public Health*. 2018;108(1):137–142. 10.2105/AJPH.2017.304050 29161058 PMC5719676

[113] Onaemo VN, Fawehinmi TO, D’Arcy C. Comorbid cannabis use disorder with major depression and generalized anxiety disorder: a systematic review with meta-analysis of nationally representative epidemiological surveys. *J Affect Disord*. 2021;281:467–475. 10.1016/j.jad.2020.12.043 33360749

[114] Palacios-Ceña D, Jiménez-Trujillo I, Hernández-Barrera V, Lima Florencio L, Carrasco-Garrido P. Time trends in the co-use of cannabis and the misuse of tranquilizers, sedatives and sleeping pills among young adults in Spain between 2009 and 2015. *Int J Environ Res Public Health*. 2019;16(18):3423. 10.3390/ijerph16183423 31540173 PMC6765996

[115] Rognli EB, Bramness JG, von Soest T. Cannabis use in early adulthood is prospectively associated with prescriptions of antipsychotics, mood stabilizers, and antidepressants. *Acta Psychiatr Scand*. 2020;141(2):149–156. 10.1111/acps.13104 31560790

[116] Wilson J, Mills K, Freeman TP, Sunderland M, Visontay R, Marel C. Weeding out the truth: a systematic review and meta-analysis on the transition from cannabis use to opioid use and opioid use disorders, abuse or dependence. *Addiction.* 2022;117(2):284–298. 10.1111/add.15581 34264545

[117] Hindocha C, Freeman TP, Curran HV. Anatomy of a joint: comparing self-reported and actual dose of cannabis and tobacco in a joint, and how these are influenced by controlled acute administration. *Cannabis Cannabinoid Res.* 2017;2(1):217–223. 10.1089/can.2017.0024 29082319 PMC5628568

[118] Freemon FR, Al-Marashi MS. Long-term changes in the sleep of normal volunteers administered multiple doses of delta-9-tetrahydrocannabinol. *Drug Alcohol Depend*. 1977;2(1):39–43. 10.1016/0376-8716(77)90019-9

[119] Freemon FR . The effect of chronically administered delta-9-tetrahydrocannabinol upon the polygraphically monitored sleep of normal volunteers. *Drug Alcohol Depend*. 1982;10(4):345–353. 10.1016/0376-8716(82)90036-9 6299682

[120] Freemon FR . The effect of δ9-tetrahydrocannabinol on sleep. *Psychopharmacologia.* 1974;35(1):39–44. 10.1007/BF00421564

[121] Walsh JH, Maddison KJ, Rankin T, et al. Treating insomnia symptoms with medicinal cannabis: a randomized, crossover trial of the efficacy of a cannabinoid medicine compared with placebo. *Sleep.* 2021;44(11). 10.1093/sleep/zsab149 PMC8598183 34115851

[122] Pivik RT, Zarcone V, Dement WC, Hollister LE. Delta-9-tetrahydrocannabinol and synhexl: effects on human sleep patterns. *Clin Pharmacol Ther*. 1972;13(3):426–435. 10.1002/cpt1972133426 4337346

[123] Tassinari CA, Ambrosetto G, Peraita-Adrado MR, Gastaut H. The neuropsychiatric syndrome of Δ9-tetrahydrocannabinol and cannabis intoxication in naive subjects. In: Nahas GG, Sutin KM, Harvey D, Agurell S, Pace N, Cancro R, eds. Marihuana and Medicine. Totowa, NJ, USA: Humana Press; 1976:649–664. 10.1007/978-1-59259-710-9_64

[124] Althoff MD, Kinney GL, Aloia MS, Sempio C, Klawitter J, Bowler RP. The impact of cannabis use proximal to sleep and cannabinoid metabolites on sleep architecture. *J Clin Sleep Med*. 2024;20(10):1615–1625. 10.5664/jcsm.11212 PMC11446118 38804689

[125] Park SH, Weber F. Neural and homeostatic regulation of REM sleep. *Front Psychol*. 2020;11:1662. 10.3389/fpsyg.2020.01662 32793050 PMC7385183

[126] de Almeida CMO, Brito MMC, Bosaipo NB, et al. Cannabidiol for rapid eye movement sleep behavior disorder. *Mov Disord*. 2021;36(7):1711–1715. 10.1002/mds.28577 33754375

[127] Cappuccio FP, D’Elia L, Strazzullo P, Miller MA. Sleep duration and all-cause mortality: a systematic review and meta-analysis of prospective studies. *Sleep.* 2010;33(5):585–592. 10.1093/sleep/33.5.585 20469800 PMC2864873

[128] Kurina LM, McClintock MK, Chen JH, Waite LJ, Thisted RA, Lauderdale DS. Sleep duration and all-cause mortality: a critical review of measurement and associations. *Ann Epidemiol*. 2013;23(6):361–370. 10.1016/j.annepidem.2013.03.015 23622956 PMC3660511

[129] García-Perdomo HA, Zapata-Copete J, Rojas-Cerón CA. Sleep duration and risk of all-cause mortality: a systematic review and meta-analysis. *Epidemiol Psychiatr Sci*. 2018;28(5):578–588. 10.1017/S2045796018000379 30058510 PMC6998920

[130] Sasaki Y, Fukuda K, Takeuchi T, Inugami M, Miyasita A. Sleep onset REM period appearance rate is affected by REM propensity in circadian rhythm in normal nocturnal sleep. *Clin Neurophysiol*. 2000;111(3):428–433. 10.1016/S1388-2457(99)00254-0 10699402

[131] Carskadon MA, Dement WC. Chapter 2—normal human sleep: an overview. In: Kryger MH, Roth T, Dement WC, eds. Principles and Practice of Sleep Medicine. 6th ed. Philadelphia, PA, USA: Elsevier Saunders; 2017:15–24. 10.1016/B978-0-323-24288-2.00002-7

[132] Clark I, Landolt HP. Coffee, caffeine, and sleep: a systematic review of epidemiological studies and randomized controlled trials. *Sleep Med Rev*. 2017;31:70–78. 10.1016/j.smrv.2016.01.006 26899133

[133] King DD, Gill CJ, Cadieux CS, Singh N. The role of stigma in cannabis use disclosure: an exploratory study. *Harm Reduct J*. 2024;21(1):21. 10.1186/s12954-024-00929-8 38273362 PMC10811895

[134] Piper BJ, DeKeuster RM, Beals ML, et al. Substitution of medical cannabis for pharmaceutical agents for pain, anxiety, and sleep. *J Psychopharmacol (Oxf)*. 2017;31(5):569–575. 10.1177/0269881117699616 28372506

[135] Corroon JM Jr, Mischley LK, Sexton M. Cannabis as a substitute for prescription drugs—a cross-sectional study. *J Pain Res*. 2017;10:989–998. 10.2147/JPR.S134330 28496355 PMC5422566

