# Supplementary figures and images for: Chronic cannabis use and sleep architecture: a cross-sectional analysis of polysomnography outcomes in a sleep-clinic cohort
Source: Sleep. 2025 Dec 18;49(5):zsaf396. doi: 10.1093/sleep/zsaf396 (PMC13163167; doi:10.1093/sleep/zsaf396)

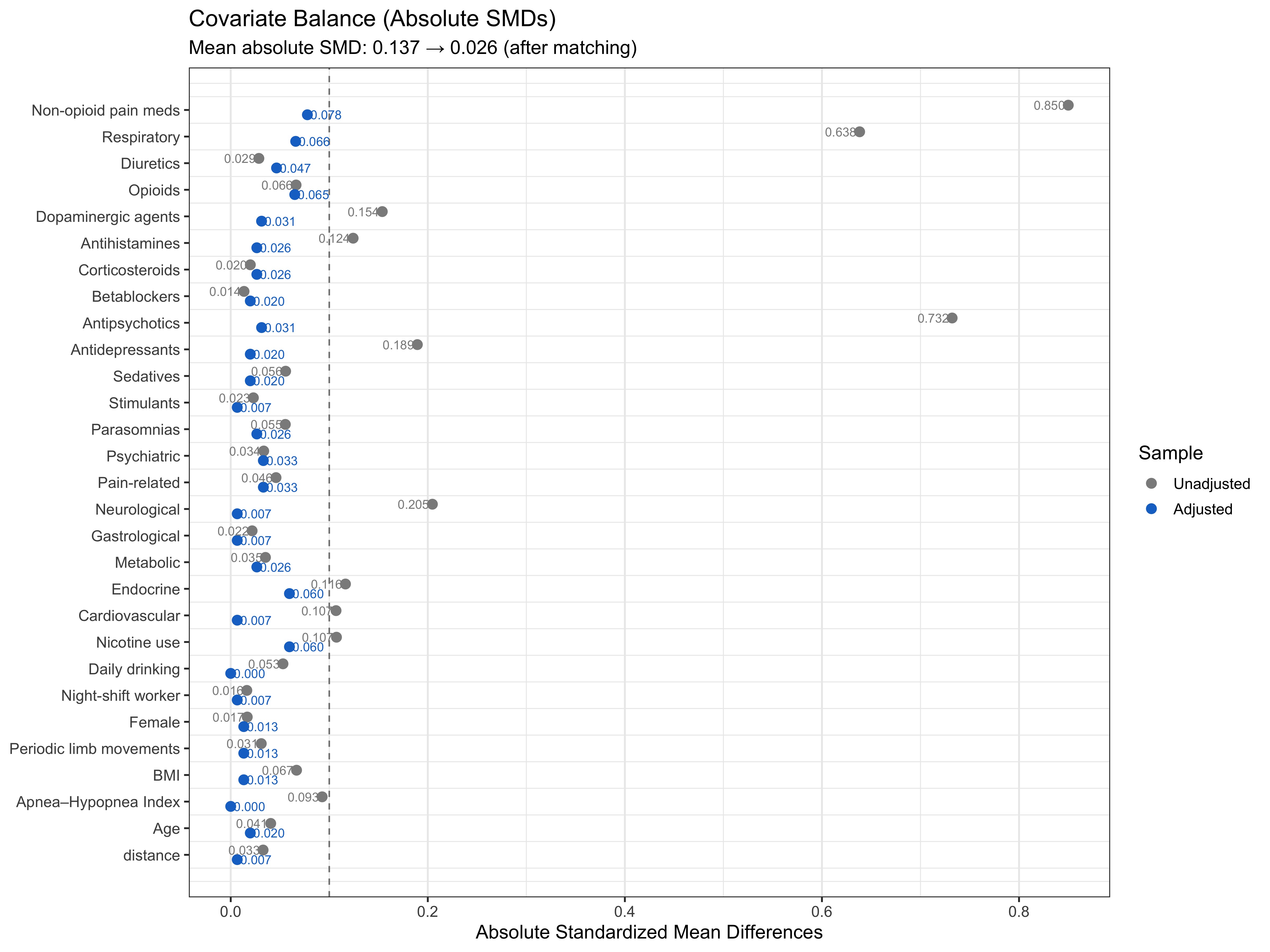

Supplement: Figure_S1_Love_plot_zsaf396 [file figure_s1_love_plot_zsaf396.jpeg]
