# Supplementary material for: Chronic cannabis use and sleep architecture: a cross-sectional analysis of polysomnography outcomes in a sleep-clinic cohort
Source: Sleep. 2025 Dec 18;49(5):zsaf396. doi: 10.1093/sleep/zsaf396 (PMC13163167; doi:10.1093/sleep/zsaf396)
Supplement: SLEEP_Supplementary_Materials_for_publication_zsaf396 [file sleep_supplementary_materials_for_publication_zsaf396.docx]

**Title:** Chronic cannabis use and sleep architecture

**Subtitle:** A cross-sectional analysis of polysomnography outcomes in a sleep-clinic cohort

**Authors and affiliations:** Velzeboer, Rob^1,2^; Wei, Sabrina^1,3^; Lai, W.K. Wayne^1,4^

^1^ Clinical Research Department, Tranq Sleep Care, Kelowna, BC, Canada

^2^ Department of Interdisciplinary Studies, Faculty of Arts, University of British Columbia, Vancouver, BC, Canada

^3^ Department of Psychology, Faculty of Arts, University of British Columbia, Vancouver, BC, Canada

^4^ Department of Medicine – Neurology, Faculty of Medicine, University of British Columbia, Vancouver, BC, Canada

**Corresponding Author:** Rob Velzeboer, Department of Interdisciplinary Studies, University of British Columbia, Vancouver, BC, Canada. Email: [rob.velzeboer@ubc.ca](mailto:rob.velzeboer@ubc.ca).

Table of Contents

[1. Variable transformation and regression model 2](#_Toc216180036)

[2. Correlation matrix 3](#_Toc216180037)

[**Table S1.** Correlation matrix 3](#_Toc216180038)

[3. Primary analysis model outcomes 4](#_Toc216180039)

[**Table S2**. Primary regression model with back-transformed coefficients 4](#_Toc216180040)

[4. Primary analysis model diagnostics 5](#_Toc216180041)

[**Table S3**. Primary model diagnostics 5](#_Toc216180042)

[**Table S4.** Influence-adjusted model for sleep stages 5](#_Toc216180043)

[5. Sensitivity models 6](#_Toc216180044)

[**Table S5A**. Outlier exclusion 6](#_Toc216180045)

[**Table S5B**. Minimal specifications 6](#_Toc216180046)

[**Table S5C.** Block exclusion 6](#_Toc216180047)

[**Table S5D**. Propensity Score Matching 7](#_Toc216180048)

[**Figure S1**. Love Plot 8](#_Toc216180049)

[**Table S6.** Sleep staging effects when holding macro-sleep constant 8](#_Toc216180050)

[6. Exploratory analyses 9](#_Toc216180051)

[**Table S7A**. Analyses stratified by sex 9](#_Toc216180052)

[**Table S7B**. Analyses stratified by apnea hypopnea severity index 9](#_Toc216180053)

[**Table S7C**. Analyses stratified by age 10](#_Toc216180054)

[**Table S8**. Analyses stratified by sex 10](#_Toc216180055)

[**Table S9.** Cannabis use patterns 11](#_Toc216180056)

[**Table S10.** Dose-response analyses 12](#_Toc216180057)

# **Variable transformation and regression model**

To estimate the association between cannabis use and objective sleep architecture outcomes, we employed a covariate-adjusted regression framework tailored to each outcome’s distributional properties and structural characteristics. Cannabis use (treatment = 1 vs. 0) was the primary independent variable of interest. All models adjusted for the same set of covariates.

For continuous outcomes with moderate-to-severe right-skew (TST, SOL, WASO, REM latency, and sleep efficiency), generalised linear models were fitted using ordinary least squares (OLS) estimation on log-transformed outcome variables (log[x + 1]) to stabilise variance and improve model fit. The regression specification was: $\log\left( Y_{i}+1 \right)=\beta_{0}+\beta_{1}\cdot\text{Cannabis}_{i}+\sum_{k=2}^{K} \beta_{k}X_{ik}+\varepsilon_{i}$, where Y*_i_* denotes the outcome for individual i, $\text{Cannabis}_{i}\in\{0,1\}$ is the treatment indicator, and $Xik$ represents covariates. Heteroskedasticity-consistent (HC3) standard errors were used for all models via the R sandwich package to account for residual non-constant variance.

Proportional outcomes N1% and N2% were modeled using quasibinomial generalised linear models with a logit link. For N3% and REM%, which had a substantial proportion of zero values, we implemented a two-part modelling strategy. First, we estimated a binary logistic regression to assess the likelihood of a zero-valued outcome: $\Pr\left( Y_{i}=0 \right)=\frac{1}{1+\exp\left( -\left[ \alpha_{0}+\alpha_{1}\cdot\text{Cannabis}_{i}+\sum_{k=2}^{K} \alpha_{k}X_{ik} \right] \right)}$. Second, among observations with non-zero values, we fitted a separate OLS model on logit-transformed values, estimating the conditional effect of cannabis use on the outcome: $Y_{i}\mid\left( Y_{i}>0 \right)=\gamma_{0}+\gamma_{1}\cdot\text{Cannabis}_{i}+\sum_{k=2}^{K} \gamma_{k}X_{ik}+\varepsilon_{i}$. Both model components were interpreted jointly: the logistic part evaluated the probability of complete absence of a sleep stage, while the linear part estimated the magnitude of difference among those with measurable values.

Nicotine use was coded 0 (never used), 0.5 (former use; > last 3 months), to 1 (active use). Daily drinking was coded as 0 for those who did not report daily alcohol consumption and 1 for those who do.

# **Correlation matrix**

| **Table S1.** Correlation matrix | | | | | | | | | | |
| --- | --- | --- | --- | --- | --- | --- | --- | --- | --- | --- |
|  | TST | SE | SOL | WASO | REML | N1 | N2 | N3 | REM | Avg |
| age | -0.3 | -0.36 | 0.03 | 0.38 | 0.03 | 0.14 | 0.15 | -0.26 | -0.12 | 0.197 |
| ahiseverity | -0.12 | -0.16 | -0.06 | 0.2 | 0.18 | 0.39 | 0.07 | -0.31 | -0.26 | 0.194 |
| sex | 0.07 | 0.06 | 0.2 | -0.16 | 0.11 | -0.23 | -0.04 | 0.27 | 0.01 | 0.128 |
| antidepressants | 0.1 | 0.11 | 0.14 | -0.19 | 0.31 | -0.01 | -0.01 | 0.09 | -0.11 | 0.119 |
| cardiovascularcondition | -0.15 | -0.19 | -0.03 | 0.22 | 0.04 | 0.13 | 0.07 | -0.16 | -0.1 | 0.121 |
| psychcondition | 0.14 | 0.16 | 0.07 | -0.21 | 0.19 | -0.05 | -0.05 | 0.14 | -0.03 | 0.116 |
| diuretic | -0.11 | -0.15 | -0.01 | 0.16 | 0.08 | 0.07 | 0.05 | -0.05 | -0.13 | 0.090 |
| plms | -0.11 | -0.12 | 0.02 | 0.11 | 0.08 | 0.1 | 0.07 | -0.13 | -0.08 | 0.091 |
| bmi | -0.01 | 0.01 | 0 | -0.01 | 0.1 | 0.07 | 0.06 | -0.05 | -0.15 | 0.051 |
| respiratorycondition | -0.06 | -0.06 | -0.01 | 0.06 | 0.03 | -0.01 | 0.04 | 0 | -0.07 | 0.038 |
| betablockers | -0.07 | -0.07 | -0.05 | 0.09 | -0.05 | 0.04 | 0.01 | -0.06 | -0.01 | 0.050 |
| stimulants | 0.08 | 0.07 | 0.01 | -0.08 | 0.08 | -0.01 | -0.05 | 0.09 | -0.03 | 0.056 |
| gastrocondition | -0.05 | -0.06 | 0.07 | 0.03 | 0.08 | -0.05 | 0.04 | 0.03 | -0.04 | 0.050 |
| endocrinecondition | -0.03 | -0.05 | 0.02 | 0.04 | 0.05 | -0.04 | 0.01 | 0.06 | -0.05 | 0.039 |
| dailydrinking | -0.07 | -0.06 | -0.02 | 0.06 | -0.02 | 0.08 | -0.01 | -0.08 | 0 | 0.044 |
| metaboliccondition | -0.05 | -0.07 | 0.02 | 0.07 | 0.05 | 0.02 | 0.08 | -0.07 | -0.09 | 0.058 |
| neurologicalcondition | -0.02 | -0.03 | -0.01 | 0.03 | 0.04 | 0.01 | -0.04 | 0.05 | -0.01 | 0.027 |
| sedatives | 0 | 0 | 0.11 | -0.05 | 0.1 | -0.02 | 0.03 | 0.03 | -0.07 | 0.046 |
| antipsychotics | 0.03 | 0.07 | 0.03 | -0.09 | 0.06 | -0.05 | 0 | 0.04 | 0.01 | 0.042 |
| corticosteroids | -0.01 | -0.02 | 0.01 | 0.01 | 0.04 | 0 | 0.03 | -0.01 | -0.05 | 0.020 |
| nicotine | 0 | 0.04 | 0.01 | -0.06 | -0.01 | 0.1 | -0.05 | -0.06 | 0.01 | 0.038 |
| paincondition | -0.02 | -0.01 | 0.05 | -0.01 | 0.07 | -0.05 | 0.02 | 0.04 | -0.02 | 0.032 |
| parasomnia | 0.02 | 0.05 | -0.04 | -0.03 | 0.02 | 0.03 | -0.04 | 0.01 | 0.02 | 0.029 |
| dopaminergic | 0.07 | 0.03 | 0 | -0.02 | 0.08 | 0.02 | 0 | 0 | -0.03 | 0.028 |
| nightshifts | 0.06 | 0.07 | -0.03 | -0.06 | 0.01 | -0.03 | 0 | 0.02 | 0.02 | 0.033 |
| opioid | 0.01 | 0.03 | 0 | -0.03 | 0.06 | 0.04 | 0.02 | -0.02 | -0.06 | 0.030 |
| nonopioidpainmeds | 0.02 | 0.03 | 0.03 | -0.05 | 0.05 | 0 | 0.03 | -0.01 | -0.04 | 0.029 |
| antihistamines | 0.02 | 0 | 0 | 0.01 | 0.02 | -0.05 | 0 | 0.07 | -0.02 | 0.021 |

| **Primary analysis model outcomes****Table S2**. Primary regression model with back-transformed coefficients | | | | |  |  |  |
| --- | --- | --- | --- | --- | --- | --- | --- |
|  |  | Model | β (95% CI) | p (q) | Interpretable coefficient | % change (CI) |  |
| **Sleep quantity and efficiency** | | |  |  |  |  | |
|  | Total sleeping time | OLS (log-transformed) | -0.034 (-0.063‚ -0.005) | **0.023 (0.062)** | -11.76 min (-22.26‚ -0.92) | -3.3% (-6.3‚ -0.3%) |  |
|  | Sleep efficiency | Quasibinomial GLM (logit) | -0.160 (-0.270‚ -0.048) | **0.005 (0.018)** | -2.91 pp (-5.13‚ -0.81) | -3.8% (-6.6‚ -1.0%) |  |
| **Transitions and fragmentation** | | |  |  |  |  | |
|  | Sleep onset latency | OLS (log-transformed) | 0.012 (-0.165‚ 0.189) | 0.894 (0.894) | 0.31 min (-4.25‚ 5.83) | 1.2% (-16.4‚ 22.5%) |  |
|  | Wake after sleep onset | OLS (log-transformed) | 0.191 (0.070‚ 0.311) | **0.002 (0.012)** | 16.61 min (5.31‚ 29.41) | 21.0% (6.7‚ 37.2%) |  |
|  | REM latency | OLS (log-transformed) | 0.028 (-0.081‚ 0.136) | 0.619 (0.783) | 4.65 min (-13.47‚ 24.91) | 2.8% (-8.1‚ 15.0%) |  |
| **Sleep staging** | |  |  |  |  |  | |
|  | N1% | Quasibinomial GLM (logit) | 0.223 (0.080‚ 0.364) | **0.002 (0.012)** | 2.77 pp (0.32‚ 5.56) | 21.0% (2.4‚ 42.2%) |  |
|  | N2% | Quasibinomial GLM (logit) | -0.038 (-0.138‚ 0.062) | 0.458 (0.783) | -0.91 pp (-3.74‚ 1.86) | -1.5% (-6.2‚ 3.1%) |  |
|  | N3% | OLS (logit-transformed) | -0.149 (-0.378‚ 0.080) | 0.202 (0.445) | -1.33 pp (-3.20‚ 0.97) | -12.6% (-30.3‚ 9.1%) |  |
|  | N3 presence | Logistic regression | -0.116 (-0.635‚ 0.429) | 0.668 (0.783) | -1.57 pp (-10.80‚ 4.86) | -1.9% (-12.7‚ 5.7%) |  |
|  | REM% | OLS (logit-transformed) | -0.025 (-0.159‚ 0.109) | 0.712 (0.783) | -0.29 pp (-1.63‚ 1.17) | -2.2% (-12.0‚ 8.6%) |  |
|  | REM presence | Logistic regression | -0.237 (-1.147‚ 0.821) | 0.632 (0.783) | -1.08 pp (-10.04‚ 2.39) | -1.1% (-10.5‚ 2.5%) |  |

# **Primary analysis model diagnostics**

| **Table S3**. Primary model diagnostics | | | |  |  | | |  | | |  | | |  | | |  | | |  | |  |  |  |  |  |  |
| --- | --- | --- | --- | --- | --- | --- | --- | --- | --- | --- | --- | --- | --- | --- | --- | --- | --- | --- | --- | --- | --- | --- | --- | --- | --- | --- | --- |
|  | Model | n | RESET | BP | φ | MF | AUC | | HL | Env | | MASR | outl | | MCD | C n | | Hat n | Max VIF | |  |  |  |  |  |  |  |
| TST | OLS (log-transformed) | 1449 | 0.77 | 0.24 | NA | NA | NA | | NA | 45.30 | | 2.86 | 0 | | 0.01 | 78 | | 80 | 1.67 | |  |  |  |  |  |  |  |
| Sleep efficiency | Quasibinomial GLM (logit) | 1449 | 0.28 | 0.79 | NA | NA | NA | | NA | 73.60 | | 3.25 | 0 | | 0.02 | 80 | | 90 | 1.65 | |  |  |  |  |  |  |  |
| SOL | OLS (log-transformed) | 1449 | 0.32 | 0.15 | NA | NA | NA | | NA | 99.10 | | 3.50 | 0 | | 0.01 | 88 | | 80 | 1.67 | |  |  |  |  |  |  |  |
| WASO | OLS (log-transformed) | 1449 | 0.49 | 0.03 | NA | NA | NA | | NA | 98.60 | | 4.32 | 1 | | 0.02 | 72 | | 80 | 1.67 | |  |  |  |  |  |  |  |
| REM latency | OLS (log-transformed) | 1388 | 0.06 | 0.87 | NA | NA | NA | | NA | 60.70 | | 6.40 | 4 | | 0.04 | 67 | | 75 | 1.67 | |  |  |  |  |  |  |  |
| N1% | Quasibinomial GLM (logit) | 1445 | 1.00 | NA | 0.08 | NA | NA | | NA | NA | | NA | NA | | 0.07 | 101 | | 120 | 1.67 | |  |  |  |  |  |  |  |
| N2% | Quasibinomial GLM (logit) | 1449 | 1.00 | NA | 0.07 | NA | NA | | NA | NA | | NA | NA | | 0.03 | 89 | | 79 | 1.67 | |  |  |  |  |  |  |  |
| N3% | Logistic regression | 1449 | 1.00 | NA | 1.05 | 0.14 | 0.76 | | 0.54 | NA | | NA | NA | | 0.01 | 133 | | 186 | 1.66 | |  |  |  |  |  |  |  |
| N3 presence | OLS (logit-transformed) | 1227 | 1.00 | NA | NA | NA | NA | | NA | NA | | NA | NA | | 0.01 | 73 | | 76 | 1.69 | |  |  |  |  |  |  |  |
| REM% | Logistic regression | 1449 | 0.83 | NA | 0.83 | 0.18 | 0.83 | | 0.24 | NA | | NA | NA | | 0.06 | 70 | | 204 | 1.88 | |  |  |  |  |  |  |  |
| REM presence | OLS (logit-transformed) | 1387 | 0.50 | NA | NA | NA | NA | | NA | NA | | NA | NA | | 0.03 | 80 | | 76 | 1.67 | |  |  |  |  |  |  |  |
| RESET = Ramsey RESET test (p-value); BP = Breusch-Pagan test (p-value); φ = dispersion ratio (Pearson χ² / df); MF = McFadden Pseudo-R²; AUC = Area Under ROC Curve; HL = Hosmer-Lemeshow (p-value); Env =  % of studentised residuals within half-normal envelope; MASR = Max Absolute Studentised Residual; outl = studentised residuals; MCD = Maximum Cook's Distance; C n = observations with Cook's D > 4/n; Hat n =  observations with leverage > 2k/n; Max VIF = Maximum Variance Inflation Factor | | | | | | | | | | | | | | | | | | | | | | | | | | | |

| **Table S4.** Influence-adjusted model for sleep stages | | |  | | | | | | | | | | | |  |  |  |  |  |  |  |  |  |  |  |  |
| --- | --- | --- | --- | --- | --- | --- | --- | --- | --- | --- | --- | --- | --- | --- | --- | --- | --- | --- | --- | --- | --- | --- | --- | --- | --- | --- |
|  | Model | β (95% CI) | p (q) | n | RESET | φ | MF | AUC | HL | MCD | C n | Hat n | Max VIF |  |  |  |  |  |  |  |  |  |  |  |  |  |
| N1% | Quasibinomial GLM (logit) | **0.189 (0.047‚ 0.329)** | **0.009 (0.032)** | 1266 | 1.00 | 0.05 | NA | NA | NA | 0.01 | 77 | 92 | 1.70 |  |  |  |  |  |  |  |  |  |  |  |  |  |
| N2% | Quasibinomial GLM (logit) | -0.038 (-0.138‚ 0.062) | 0.458 (0.719) | 1449 | 1.00 | 0.07 | NA | NA | NA | 0.03 | 89 | 79 | 1.67 |  |  |  |  |  |  |  |  |  |  |  |  |  |
| N3% | Logistic regression | 1.076 (-0.436‚ 3.100) | 0.217 (0.397) | 1178 | 0.81 | 0.73 | 0.41 | 0.93 | 0.72 | 0.03 | 71 | 186 | 1.98 |  |  |  |  |  |  |  |  |  |  |  |  |  |
| N3 presence | OLS (logit-transformed) | -0.150 (-0.349‚ 0.048) | 0.138 (0.304) | 1088 | 1.00 | NA | NA | NA | NA | 0.01 | 60 | 55 | 1.69 |  |  |  |  |  |  |  |  |  |  |  |  |  |
| REM presence | OLS (logit-transformed) | -0.015 (-0.115‚ 0.085) | 0.764 (0.934) | 1241 | 1.00 | NA | NA | NA | NA | 0.01 | 73 | 73 | 1.68 |  |  |  |  |  |  |  |  |  |  |  |  |  |
| RESET = Ramsey RESET test (p-value); φ = dispersion ratio (Pearson χ² / df); MF = McFadden Pseudo-R²; AUC = Area Under ROC Curve; HL = Hosmer-Lemeshow (p-value); MCD = Maximum Cook's Distance; C n =  observations with Cook's D > 4/n; Hat n = observations with leverage > 2k/n; Max VIF = Maximum Variance Inflation Factor | | | | | | | | | | | | | | | | | | | | | | | | | | |

# **Sensitivity models**

| **Table S5A**. Outlier exclusion | |  |  |  |  |
| --- | --- | --- | --- | --- | --- |
|  | **±3SD winsor** |  |  | **±3SD trimmed** |  |
|  | β (95% CI) | p (q) |  | β (95% CI) | p (q) |
| TST | **-0.034 (-0.063‚ -0.005)** | **0.023 (0.062)** | | -0.028 (-0.058‚ 0.002) | 0.066 (0.198) |
| Sleep efficiency | **-0.160 (-0.271‚ -0.048)** | **0.005 (0.018)** | | **-0.143 (-0.259‚ -0.027)** | **0.016 (0.089)** |
| SOL | 0.011 (-0.164‚ 0.186) | 0.904 (0.904) | | 0.045 (-0.138‚ 0.227) | 0.632 (0.772) |
| WASO | **0.190 (0.070‚ 0.310)** | **0.002 (0.018)** | | **0.180 (0.054‚ 0.306)** | **0.005 (0.056)** |
| REM latency | 0.027 (-0.081‚ 0.136) | 0.622 (0.786) | | 0.033 (-0.078‚ 0.145) | 0.556 (0.765) |
| N1% | **0.199 (0.062‚ 0.336)** | **0.004 (0.018)** | | 0.130 (-0.011‚ 0.272) | 0.072 (0.198) |
| N2% | -0.033 (-0.132‚ 0.067) | 0.521 (0.786) | | 0.016 (-0.083‚ 0.115) | 0.752 (0.827) |
| N3 presence | -0.116 (-0.646‚ 0.414) | 0.668 (0.786) | | -0.178 (-0.757‚ 0.400) | 0.546 (0.765) |
| N3% | -0.147 (-0.375‚ 0.081) | 0.207 (0.455) | | -0.118 (-0.356‚ 0.120) | 0.331 (0.727) |
| REM presence | -0.237 (-1.207‚ 0.732) | 0.632 (0.786) | | NA | NA |
| REM% | -0.025 (-0.159‚ 0.109) | 0.714 (0.786) | | -0.054 (-0.190‚ 0.082) | 0.437 (0.765) |
|  |  |  |  |  |  |
| **Table S5B**. Minimal specifications | | |  |  |  |
|  | **Minimal #1 (3 variable)** | |  | **Minimal #2 (7 variable)** | |
|  | β (95% CI) | p (q) |  | β (95% CI) | p (q) |
| TST | -0.027 (-0.054‚ 0.001) | 0.055 (0.207) | | **-0.031 (-0.059‚ -0.004)** | **0.027 (0.088)** |
| Sleep efficiency | -0.102 (-0.208‚ 0.004) | 0.060 (0.207) | | **-0.122 (-0.228‚ -0.015)** | **0.025 (0.088)** |
| SOL | 0.040 (-0.130‚ 0.209) | 0.647 (0.791) | | 0.020 (-0.149‚ 0.189) | 0.817 (0.987) |
| WASO | 0.095 (-0.020‚ 0.210) | 0.105 (0.232) | | **0.126 (0.011‚ 0.241)** | **0.032 (0.088)** |
| REM latency | 0.030 (-0.078‚ 0.137) | 0.586 (0.791) | | -0.001 (-0.104‚ 0.103) | 0.987 (0.987) |
| N1% | **0.254 (0.118‚ 0.390)** | **0.000 (0.003)** | | **0.257 (0.121‚ 0.393)** | **0.000 (0.002)** |
| N2% | -0.047 (-0.142‚ 0.047) | 0.326 (0.512) | | -0.052 (-0.147‚ 0.044) | 0.289 (0.454) |
| N3 presence | -0.313 (-0.805‚ 0.179) | 0.213 (0.390) | | -0.324 (-0.822‚ 0.174) | 0.202 (0.370) |
| N3% | -0.197 (-0.415‚ 0.020) | 0.075 (0.207) | | -0.210 (-0.428‚ 0.009) | 0.060 (0.133) |
| REM presence | -0.043 (-0.941‚ 0.855) | 0.926 (0.926) | | 0.076 (-0.837‚ 0.989) | 0.870 (0.987) |
| REM% | -0.015 (-0.144‚ 0.115) | 0.822 (0.904) | | -0.004 (-0.132‚ 0.124) | 0.949 (0.987) |
|  |  |  |  |  |  |
| **Table S5C.** Block exclusion | |  |  |  |  |
|  | **Medication exclusion** |  |  | **Morbidity exclusion** |  |
|  | β (95% CI) | p (q) |  | β (95% CI) | p (q) |
| TST | **-0.034 (-0.063‚ -0.005)** | **0.021 (0.057)** | | **-0.031 (-0.060‚ -0.003)** | **0.033 (0.091)** |
| Sleep efficiency | **-0.157 (-0.267‚ -0.046)** | **0.006 (0.021)** | | **-0.147 (-0.257‚ -0.036)** | **0.009 (0.035)** |
| SOL | 0.026 (-0.151‚ 0.203) | 0.774 (0.795) | | 0.011 (-0.165‚ 0.187) | 0.904 (0.904) |
| WASO | **0.177 (0.057‚ 0.297)** | **0.004 (0.021)** | | **0.178 (0.059‚ 0.297)** | **0.003 (0.019)** |
| REM latency | 0.020 (-0.091‚ 0.131) | 0.721 (0.795) | | 0.028 (-0.080‚ 0.136) | 0.610 (0.884) |
| N1% | **0.211 (0.068‚ 0.353)** | **0.004 (0.021)** | | **0.213 (0.071‚ 0.355)** | **0.003 (0.019)** |
| N2% | -0.035 (-0.134‚ 0.064) | 0.490 (0.795) | | -0.034 (-0.134‚ 0.065) | 0.496 (0.884) |
| N3 presence | -0.168 (-0.685‚ 0.349) | 0.524 (0.795) | | -0.124 (-0.650‚ 0.401) | 0.643 (0.884) |
| N3% | -0.157 (-0.385‚ 0.070) | 0.175 (0.386) | | -0.150 (-0.378‚ 0.078) | 0.197 (0.433) |
| REM presence | -0.126 (-1.077‚ 0.825) | 0.795 (0.795) | | -0.145 (-1.100‚ 0.811) | 0.767 (0.904) |
| REM% | -0.021 (-0.156‚ 0.114) | 0.762 (0.795) | | -0.014 (-0.147‚ 0.120) | 0.840 (0.904) |
|  | **Medication + morbidity exclusion** | |  |  |  |
|  | β (95% CI) | p (q) |  |  |  |
| TST | **-0.029 (-0.057‚ -0.000)** | **0.047 (0.130)** | |  |  |
| Sleep efficiency | **-0.132 (-0.242‚ -0.023)** | **0.018 (0.067)** | |  |  |
| SOL | 0.045 (-0.131‚ 0.221) | 0.618 (0.744) | |  |  |
| WASO | **0.145 (0.026‚ 0.264)** | **0.017 (0.067)** | |  |  |
| REM latency | 0.051 (-0.061‚ 0.163) | 0.370 (0.679) | |  |  |
| N1% | **0.200 (0.059‚ 0.342)** | **0.005 (0.060)** | |  |  |
| N2% | -0.029 (-0.127‚ 0.069) | 0.565 (0.744) | |  |  |
| N3 presence | -0.172 (-0.681‚ 0.337) | 0.509 (0.744) | |  |  |
| N3% | -0.154 (-0.381‚ 0.073) | 0.185 (0.407) | |  |  |
| REM presence | -0.142 (-1.074‚ 0.789) | 0.764 (0.764) | |  |  |
| REM% | -0.029 (-0.164‚ 0.106) | 0.677 (0.744) | |  |  |
|  |  |  |  |  |  |
| **Table S5D**. Propensity Score Matching | | |  |  |  |
|  | **PSM-adjusted** |  |  |  |  |
|  | β (95% CI) | p (q) |  |  |  |
| TST | -0.020 (-0.058‚ 0.018) | 0.301 (0.701) | |  |  |
| Sleep efficiency | -0.139 (-0.286‚ 0.008) | 0.066 (0.394) | |  |  |
| SOL | 0.024 (-0.213‚ 0.261) | 0.843 (0.878) | |  |  |
| WASO | 0.169 (-0.004‚ 0.342) | 0.055 (0.394) | |  |  |
| REM latency | -0.012 (-0.161‚ 0.137) | 0.878 (0.878) | |  |  |
| N1% | 0.160 (-0.121‚ 0.441) | 0.264 (0.701) | |  |  |
| N2% | -0.081 (-0.232‚ 0.070) | 0.290 (0.701) | |  |  |
| N3 presence | -0.253 (-0.886‚ 0.371) | 0.428 (0.701) | |  |  |
| N3% | -0.136 (-0.450‚ 0.178) | 0.394 (0.701) | |  |  |
| REM presence | -0.419 (-1.800‚ 0.854) | 0.523 (0.701) | |  |  |
| REM% | 0.052 (-0.110‚ 0.215) | 0.526 (0.701) | |  |  |

### **Figure S1**. Love Plot

**
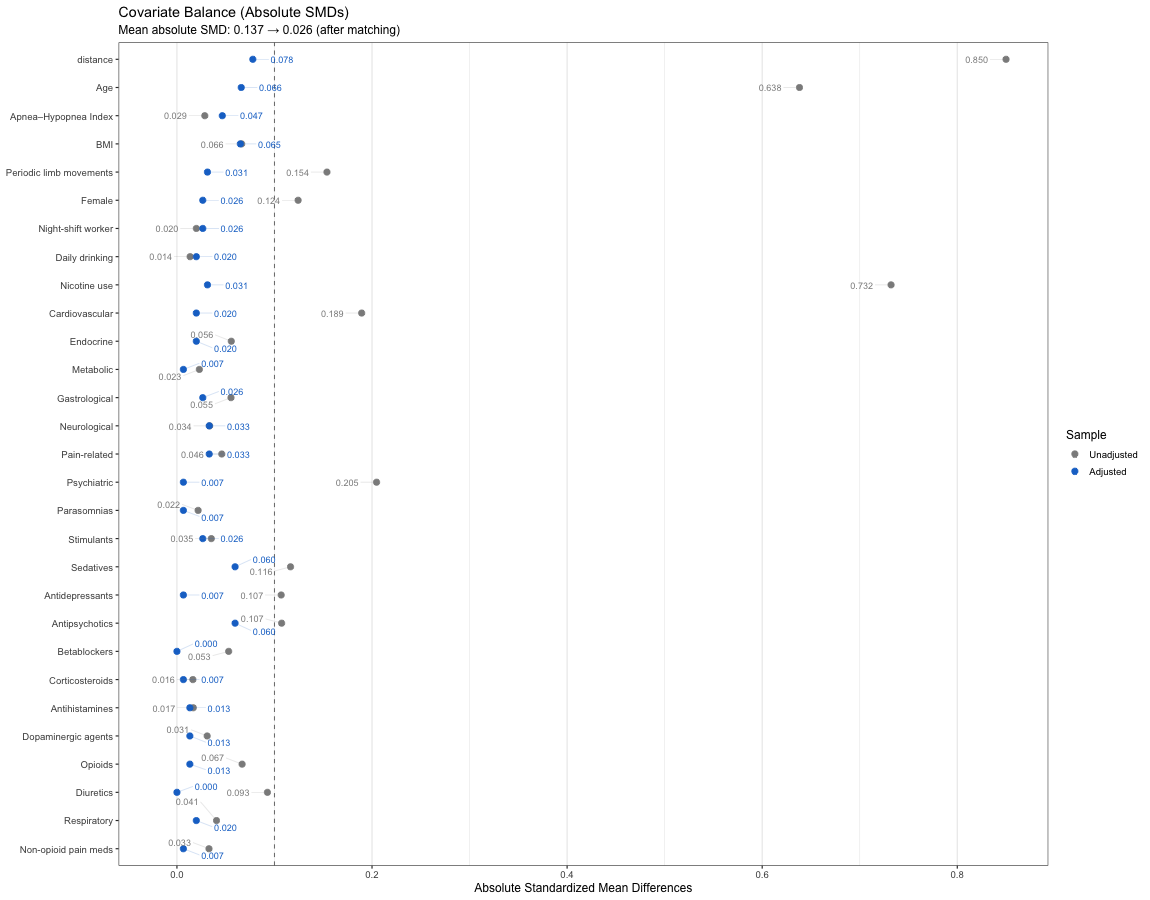
**

| **Table S6.** Sleep staging effects when holding macro-sleep constant | | | | |  |
| --- | --- | --- | --- | --- | --- |
|  | **Regression** |  |  | **PSM-adjusted** |  |
|  | β (95% CI) | p (q) |  | β (95% CI) | p (q) |
| N1% | **0.173 (0.037‚ 0.307)** | **0.012 (0.067)** | | 0.006 (-0.226‚ 0.238) | 0.958 (0.958) |
| N2% | -0.023 (-0.122‚ 0.077) | 0.654 (0.860) | | -0.018 (-0.172‚ 0.137) | 0.822 (0.901) |
| N3 presence | -0.103 (-0.622‚ 0.443) | 0.704 (0.860) | | 0.291 (-0.418‚ 1.014) | 0.423 (0.901) |
| N3% | -0.159 (-0.389‚ 0.070) | 0.173 (0.477) | | -0.096 (-0.389‚ 0.198) | 0.520 (0.901) |
| REM presence | -0.197 (-1.121‚ 0.872) | 0.694 (0.860) | | 0.500 (-1.075‚ 2.202) | 0.541 (0.901) |
| REM% | 0.012 (-0.120‚ 0.144) | 0.861 (0.861) | | -0.029 (-0.184‚ 0.127) | 0.718 (0.901) |

# **Exploratory analyses**

| **Table S7A**. Analyses stratified by sex | | | | | | | | | |  |
| --- | --- | --- | --- | --- | --- | --- | --- | --- | --- | --- |
|  |  | **Male** | |  | |  | **Female** | |  | |
|  |  | β (95% CI) | | p (q) | |  | β (95% CI) | | p (q) | |
| TST |  | **-0.049 (-0.095‚ -0.002)** | | **0.042 (0.185)** | |  | -0.026 (-0.064‚ 0.012) | | 0.182 (0.364) | |
| Sleep efficiency | | **-0.313 (-0.498‚ -0.129)** | | **0.001 (0.022)** | |  | -0.066 (-0.208‚ 0.077) | | 0.365 (0.415) | |
| SOL |  | **0.329 (0.037‚ 0.622)** | | **0.028 (0.154)** | |  | -0.166 (-0.391‚ 0.059) | | 0.148 (0.326) | |
| WASO |  | **0.286 (0.083‚ 0.488)** | | **0.006 (0.051)** | |  | 0.124 (-0.025‚ 0.274) | | 0.104 (0.254) | |
| REM latency |  | 0.154 (-0.006‚ 0.314) | | 0.059 (0.185) | |  | -0.068 (-0.221‚ 0.085) | | 0.386 (0.415) | |
| N1% |  | **0.027 (0.007‚ 0.047)** | | **0.007 (0.051)** | |  | 0.013 (-0.009‚ 0.035) | | 0.232 (0.396) | |
| N2% |  | -0.002 (-0.025‚ 0.021) | | 0.858 (0.858) | |  | -0.011 (-0.032‚ 0.009) | | 0.288 (0.396) | |
| N3 presence |  | -0.926 (-1.871‚ 0.018) | | 0.055 (0.185) | |  | 0.390 (-0.295‚ 1.074) | | 0.264 (0.396) | |
| N3% |  | -0.199 (-0.534‚ 0.136) | | 0.245 (0.396) | |  | -0.143 (-0.474‚ 0.187) | | 0.396 (0.415) | |
| REM presence |  | 0.993 (-1.151‚ 3.137) | | 0.364 (0.415) | |  | -1.103 (-2.393‚ 0.186) | | 0.094 (0.254) | |
| REM% |  | -0.122 (-0.343‚ 0.099) | | 0.279 (0.396) | |  | 0.081 (-0.087‚ 0.249) | | 0.343 (0.415) | |
|  |  |  | |  | |  |  | |  | |
| **Table S7B**. Analyses stratified by apnea hypopnea severity index | | | | | | | | | |  |
|  |  | **0-<5** | |  | |  | **5-<15** | |  | |
|  |  | β (95% CI) | | p (q) | |  | β (95% CI) | | p (q) | |
| TST |  | **-0.129 (-0.220‚ -0.038)** | | **0.006 (0.082)** | |  | -0.047 (-0.105‚ 0.012) | | 0.119 (0.451) | |
| Sleep efficiency | | **-0.585 (-0.976‚ -0.195)** | | **0.004 (0.082)** | |  | -0.197 (-0.445‚ 0.051) | | 0.120 (0.451) | |
| SOL |  | 0.276 (-0.297‚ 0.848) | | 0.348 (0.711) | |  | 0.074 (-0.324‚ 0.472) | | 0.715 (0.911) | |
| WASO |  | 0.448 (-0.010‚ 0.907) | | 0.058 (0.381) | |  | 0.183 (-0.088‚ 0.455) | | 0.187 (0.590) | |
| REM latency |  | 0.001 (-0.351‚ 0.352) | | 0.997 (0.997) | |  | -0.030 (-0.266‚ 0.205) | | 0.800 (0.911) | |
| N1% |  | 0.024 (-0.011‚ 0.060) | | 0.182 (0.590) | |  | 0.006 (-0.017‚ 0.029) | | 0.596 (0.815) | |
| N2% |  | -0.036 (-0.080‚ 0.009) | | 0.117 (0.451) | |  | 0.009 (-0.021‚ 0.040) | | 0.555 (0.787) | |
| N3 presence |  | NA | | NA | |  | -0.398 (-1.727‚ 0.931) | | 0.557 (0.787) | |
| N3% |  | -0.027 (-0.637‚ 0.582) | | 0.930 (0.978) | |  | -0.186 (-0.553‚ 0.180) | | 0.320 (0.711) | |
| REM presence |  | NA | | NA | |  | -0.450 (-3.704‚ 2.803) | | 0.786 (0.911) | |
| REM% |  | 0.060 (-0.371‚ 0.490) | | 0.787 (0.911) | |  | 0.001 (-0.291‚ 0.292) | | 0.996 (0.997) | |
|  |  |  | |  | |  |  | |  | |
|  |  |  | **15-<30** | |  | |  | **≥30** | |  |
|  |  | β (95% CI) | | p (q) | |  | β (95% CI) | | p (q) | |
| TST |  | -0.057 (-0.129‚ 0.015) | | 0.121 (0.451) | |  | -0.013 (-0.057‚ 0.031) | | 0.551 (0.787) | |
| Sleep efficiency | | **-0.317 (-0.606‚ -0.028)** | | **0.033 (0.271)** | |  | -0.079 (-0.237‚ 0.080) | | 0.332 (0.711) | |
| SOL |  | 0.154 (-0.278‚ 0.585) | | 0.486 (0.787) | |  | -0.085 (-0.344‚ 0.174) | | 0.520 (0.787) | |
| WASO |  | **0.428 (0.126‚ 0.729)** | | **0.006 (0.082)** | |  | 0.157 (-0.009‚ 0.323) | | 0.065 (0.381) | |
| REM latency |  | -0.124 (-0.403‚ 0.154) | | 0.383 (0.711) | |  | 0.094 (-0.065‚ 0.253) | | 0.249 (0.646) | |
| N1% |  | -0.003 (-0.034‚ 0.028) | | 0.844 (0.935) | |  | **0.033 (0.005‚ 0.061)** | | **0.021 (0.215)** | |
| N2% |  | 0.004 (-0.029‚ 0.038) | | 0.797 (0.911) | |  | -0.015 (-0.039‚ 0.009) | | 0.233 (0.646) | |
| N3 presence |  | 1.096 (-1.316‚ 3.507) | | 0.373 (0.711) | |  | -0.242 (-0.900‚ 0.416) | | 0.471 (0.787) | |
| N3% |  | 0.037 (-0.480‚ 0.554) | | 0.889 (0.959) | |  | -0.183 (-0.608‚ 0.242) | | 0.399 (0.711) | |
| REM presence |  | NA | | NA | |  | -0.589 (-1.827‚ 0.648) | | 0.350 (0.711) | |
| REM% |  | -0.155 (-0.421‚ 0.110) | | 0.252 (0.646) | |  | 0.032 (-0.187‚ 0.252) | | 0.772 (0.911) | |
|  |  |  | |  | |  |  | |  | |
| **Table S7C**. Analyses stratified by age | | | | | | |  |  | |  |
|  |  | **18-34** | |  | |  | **35-49** | |  | |
|  |  | β (95% CI) | | p (q) | |  | β (95% CI) | | p (q) | |
| TST |  | -0.053 (-0.120‚ 0.014) | | 0.122 (0.488) | |  | -0.028 (-0.079‚ 0.024) | | 0.292 (0.687) | |
| Sleep efficiency | | -0.273 (-0.567‚ 0.020) | | 0.070 (0.350) | |  | -0.123 (-0.337‚ 0.091) | | 0.259 (0.675) | |
| SOL |  | 0.151 (-0.265‚ 0.567) | | 0.477 (0.777) | |  | -0.004 (-0.334‚ 0.326) | | 0.980 (0.980) | |
| WASO |  | 0.290 (-0.015‚ 0.594) | | 0.064 (0.350) | |  | 0.165 (-0.074‚ 0.404) | | 0.176 (0.542) | |
| REM latency |  | 0.107 (-0.114‚ 0.328) | | 0.343 (0.720) | |  | 0.092 (-0.122‚ 0.305) | | 0.401 (0.729) | |
| N1% |  | **0.033 (0.008‚ 0.059)** | | **0.011 (0.350)** | |  | 0.018 (-0.007‚ 0.043) | | 0.163 (0.542) | |
| N2% |  | **-0.032 (-0.062‚ -0.002)** | | **0.037 (0.350)** | |  | 0.008 (-0.018‚ 0.033) | | 0.555 (0.777) | |
| N3 presence |  | -0.071 (-3.444‚ 3.302) | | 0.967 (0.980) | |  | 0.077 (-1.121‚ 1.275) | | 0.900 (0.973) | |
| N3% |  | -0.080 (-0.429‚ 0.269) | | 0.653 (0.843) | |  | -0.141 (-0.531‚ 0.250) | | 0.481 (0.777) | |
| REM presence |  | NA | | NA | |  | NA | | NA | |
| REM% |  | 0.072 (-0.228‚ 0.372) | | 0.639 (0.843) | |  | -0.221 (-0.450‚ 0.008) | | 0.059 (0.350) | |
|  |  |  | |  | |  |  | |  | |
|  |  | **50-64** | |  | |  | **≥65** | |  | |
|  |  | β (95% CI) | | p (q) | |  | β (95% CI) | | p (q) | |
| TST |  | -0.039 (-0.092‚ 0.013) | | 0.144 (0.524) | |  | 0.084 (-0.006‚ 0.173) | | 0.068 (0.350) | |
| Sleep efficiency | | **-0.201 (-0.393‚ -0.008)** | | **0.042 (0.350)** | |  | 0.256 (-0.064‚ 0.575) | | 0.117 (0.488) | |
| SOL |  | 0.011 (-0.332‚ 0.354) | | 0.950 (0.980) | |  | -0.211 (-0.684‚ 0.263) | | 0.384 (0.729) | |
| WASO |  | 0.219 (-0.000‚ 0.439) | | 0.051 (0.350) | |  | -0.151 (-0.460‚ 0.159) | | 0.341 (0.720) | |
| REM latency |  | 0.057 (-0.135‚ 0.249) | | 0.563 (0.777) | |  | -0.157 (-0.491‚ 0.178) | | 0.360 (0.720) | |
| N1% |  | 0.003 (-0.026‚ 0.032) | | 0.835 (0.973) | |  | 0.003 (-0.045‚ 0.052) | | 0.894 (0.973) | |
| N2% |  | 0.010 (-0.020‚ 0.039) | | 0.517 (0.777) | |  | -0.017 (-0.063‚ 0.030) | | 0.488 (0.777) | |
| N3 presence |  | -0.166 (-1.024‚ 0.693) | | 0.705 (0.881) | |  | 0.250 (-1.260‚ 1.759) | | 0.746 (0.904) | |
| N3% |  | -0.321 (-0.827‚ 0.185) | | 0.214 (0.611) | |  | -0.055 (-0.821‚ 0.711) | | 0.888 (0.973) | |
| REM presence |  | NA | | NA | |  | NA | | NA | |
| REM% |  | 0.064 (-0.154‚ 0.283) | | 0.563 (0.777) | |  | 0.250 (-0.194‚ 0.694) | | 0.270 (0.675) | |

| **Table S8**. Analyses stratified by sex | | | |  |
| --- | --- | --- | --- | --- |
|  | Term | β | Robust SE | p (q) |
| **TST** | **Cannabis * age** | **0.003** | **0.001** | **0.014 (0.459)** |
| **Sleep efficiency** | **Cannabis * age** | **0.008** | **0.004** | **0.043 (0.471)** |
| SOL | Cannabis * age | 0.001 | 0.008 | 0.903 (0.943) |
| WASO | Cannabis * age | -0.008 | 0.004 | 0.086 (0.571) |
| REM latency | Cannabis * age | 0.000 | 0.004 | 0.926 (0.943) |
| N1% | Cannabis * age | 0.000 | 0.001 | 0.943 (0.943) |
| N2% | Cannabis * age | 0.001 | 0.001 | 0.477 (0.773) |
| N3% | Cannabis * age | -0.015 | 0.010 | 0.120 (0.660) |
| REM% | Cannabis * age | 0.003 | 0.004 | 0.445 (0.773) |
| N3 presence | Cannabis * age | -0.020 | 0.019 | 0.289 (0.719) |
| REM presence | Cannabis * age | -0.020 | 0.054 | 0.712 (0.824) |
| TST | Cannabis * AHI | 0.000 | 0.000 | 0.583 (0.773) |
| Sleep efficiency | Cannabis * AHI | 0.001 | 0.001 | 0.550 (0.773) |
| SOL | Cannabis * AHI | -0.002 | 0.003 | 0.615 (0.773) |
| WASO | Cannabis * AHI | 0.001 | 0.002 | 0.590 (0.773) |
| REM latency | Cannabis * AHI | 0.002 | 0.002 | 0.316 (0.719) |
| N1% | Cannabis * AHI | 0.000 | 0.000 | 0.272 (0.719) |
| N2% | Cannabis * AHI | 0.000 | 0.000 | 0.353 (0.719) |
| N3% | Cannabis * AHI | 0.002 | 0.005 | 0.724 (0.824) |
| REM% | Cannabis * AHI | 0.002 | 0.002 | 0.214 (0.719) |
| N3 presence | Cannabis * AHI | -0.010 | 0.009 | 0.276 (0.719) |
| REM presence | Cannabis * AHI | -0.010 | 0.019 | 0.611 (0.773) |
| TST | Cannabis * sex | 0.014 | 0.030 | 0.633 (0.773) |
| Sleep efficiency | Cannabis * sex | -0.063 | 0.112 | 0.574 (0.773) |
| SOL | Cannabis * sex | 0.379 | 0.208 | 0.068 (0.561) |
| WASO | Cannabis * sex | -0.023 | 0.126 | 0.855 (0.940) |
| REM latency | Cannabis * sex | 0.183 | 0.129 | 0.158 (0.719) |
| N1% | Cannabis * sex | 0.020 | 0.018 | 0.248 (0.719) |
| N2% | Cannabis * sex | 0.012 | 0.017 | 0.480 (0.773) |
| N3% | Cannabis * sex | -0.244 | 0.268 | 0.361 (0.719) |
| REM% | Cannabis * sex | -0.123 | 0.116 | 0.291 (0.719) |
| **N3 presence** | **Cannabis * sex** | **-1.425** | **0.662** | **0.031 (0.471)** |
| REM presence | Cannabis * sex | 1.190 | 1.329 | 0.370 (0.719) |

| **Table S9.** Cannabis use patterns | | | | |
| --- | --- | --- | --- | --- |
|  |  |  | n | % |
|  | **Years of use (n=120)** | |  |  |
|  |  | 1-2 | 15 | 12.5% |
|  |  | 3-5 | 15 | 12.5% |
|  |  | 6-9 | 9 | 7.5% |
|  |  | 10-19 | 25 | 20.8% |
|  |  | 20-29 | 20 | 16.7% |
|  |  | 30-39 | 16 | 13.3% |
|  |  | 40+ | 20 | 16.7% |
|  | **Share of adult life use (n=120)** | | |  |
|  |  | 2 to <5% | 6 | 5.0% |
|  |  | 5 to <10% | 8 | 6.7% |
|  |  | 10 to <25% | 11 | 9.2% |
|  |  | 25 to <50% | 12 | 10.0% |
|  |  | 50 to <75% | 13 | 10.8% |
|  |  | 75 to <100% | 18 | 15.0% |
|  |  | 100% | 52 | 43.3% |
|  | **Standard THC units (n=69)** | | |  |
|  |  | 1 to <10 | 8 | 11.6% |
|  |  | 10 to <20 | 13 | 18.8% |
|  |  | 20 to <30 | 18 | 26.1% |
|  |  | 30 to <50 | 16 | 23.2% |
|  |  | 50 to <100 | 7 | 10.1% |
|  |  | 100+ | 7 | 10.1% |
|  | **Type (n=100)** | |  |  |
|  |  | Edibles | 5 | 5.0% |
|  |  | Smoked | 72 | 72.0% |
|  |  | Vaporised | 23 | 23.0% |

| **Table S10.** Dose-response analyses | | |  |  |  |
| --- | --- | --- | --- | --- | --- |
|  | **STUs** |  |  | **Use duration** |  |
|  | β (95% CI) | p (q) |  | β (95% CI) | p (q) |
| TST | -0.001 (-0.040, 0.041) | 0.958 (0.958) |  | 0.019 (-0.014, 0.051) | 0.257 (0.757) |
| Sleep efficiency | 0.040 (-0.096, 0.177) | 0.568 (0.958) |  | 0.046 (-0.074, 0.167) | 0.454 (0.757) |
| SOL | -0.211 (-0.456, 0.034) | 0.090 (0.449) |  | 0.027 (-0.183, 0.237) | 0.799 (0.895) |
| WASO | -0.029 (-0.181, 0.122) | 0.701 (0.958) |  | -0.060 (-0.209, 0.088) | 0.423 (0.757) |
| REM latency | -0.006 (-0.147, 0.135) | 0.935 (0.958) |  | -0.009 (-0.147, 0.129) | 0.895 (0.895) |
| N1% | -0.035 (-0.285, 0.216) | 0.785 (0.939) |  | -0.054 (−0.333, 0.225) | 0.703 (0.741) |
| N2% | -0.006 (-0.162, 0.150) | 0.939 (0.939) |  | 0.033 (-0.162, 0.227) | 0.741 (0.741) |
| N3 presence | NA | NA |  | NA | NA |
| N3% | 0.042 (-0.296, 0.381) | 0.806 (0.939) |  | -0.087 (-0.346, 0.171) | 0.506 (0.741) |
| REM presence | NA | NA |  | NA | NA |
| REM% | 0.015 (-0.123, 0.153) | 0.834 (0.939) |  | 0.030 (−0.069, 0.128) | 0.552 (0.741) |
